# Muscle Regeneration in Holothurians without the Upregulation of Muscle Genes

**DOI:** 10.3390/ijms232416037

**Published:** 2022-12-16

**Authors:** Vladimir A. Nizhnichenko, Alexey V. Boyko, Talia T. Ginanova, Igor Yu. Dolmatov

**Affiliations:** A.V. Zhirmunsky National Scientific Center of Marine Biology, Far Eastern Branch, Russian Academy of Sciences, Palchevsky 17, 690041 Vladivostok, Russia

**Keywords:** holothurians, regeneration, muscle system, transcription factors, matrix metalloproteinases

## Abstract

The holothurian *Eupentacta fraudatrix* is capable of fully restoring its muscles after transverse dissection. Although the regeneration of these structures is well studied at the cellular level, the molecular basis of the process remains poorly understood. To identify genes that may be involved in the regulation of muscle regeneration, the transcriptome of the longitudinal muscle band of *E. fraudatrix* has been sequenced at different time periods post-injury. An analysis of the map of biological processes and pathways has shown that most genes associated with myogenesis decrease their expression during the regeneration. The only exception is the genes united by the GO term “heart valve development”. This may indicate the antiquity of mechanisms of mesodermal structure transformation, which was co-opted into various morphogeneses in deuterostomes. Two groups of genes that play a key role in the regeneration have been analyzed: transcription factors and matrix metalloproteinases. A total of six transcription factor genes (*Ef-HOX5, Ef-ZEB2, Ef-RARB, Ef-RUNX1, Ef-SOX17,* and *Ef-ZNF318*) and seven matrix metalloproteinase genes (*Ef-MMP11, Ef-MMP13, Ef-MMP13-1, Ef-MMP16-2, Ef-MMP16-3, Ef-MMP24,* and *Ef-MMP24-1*) showing differential expression during myogenesis have been revealed. The identified genes are assumed to be involved in the muscle regeneration in holothurians.

## 1. Introduction

The ability to restore the integrity of the organism after injury, loss, or natural wear of body parts is a fundamental property of living beings. Many animals can fully or partially regenerate structures of their muscle system [1]. This process varies between species, showing specific features on the cellular and molecular levels [2]. In vertebrates, skeletal muscles are regenerated by stem cells, referred to as myosatellite cells. These are activated in response to damage, proliferate, and fuse with muscle fibers, thereby, restoring their integrity [1]. However, some vertebrate species have other mechanisms of muscle regeneration. Thus, zebrafish (*Danio rerio*) can repair the damaged heart muscle through dedifferentiation of cardiomyocytes and transdifferentiation of fibroblasts [3,4]. During the regeneration of amputated limb in amphibians, muscles are formed through dedifferentiation of multinucleated muscle cells [5].

Invertebrates also exhibit various mechanisms of muscle regeneration. For example, in the hydroid *Podocoryna carnea*, damage to the bell induces the dedifferentiation and migration of striated muscle cells that then fill the wound site and differentiate into functional muscle cells [6]. In *Hydra*, muscles are regenerated by stem cells [7,8]; in lancelets, the tail is regenerated through dedifferentiation and the oral cirri are regenerated by stem cells [8].

**Figure 1:**
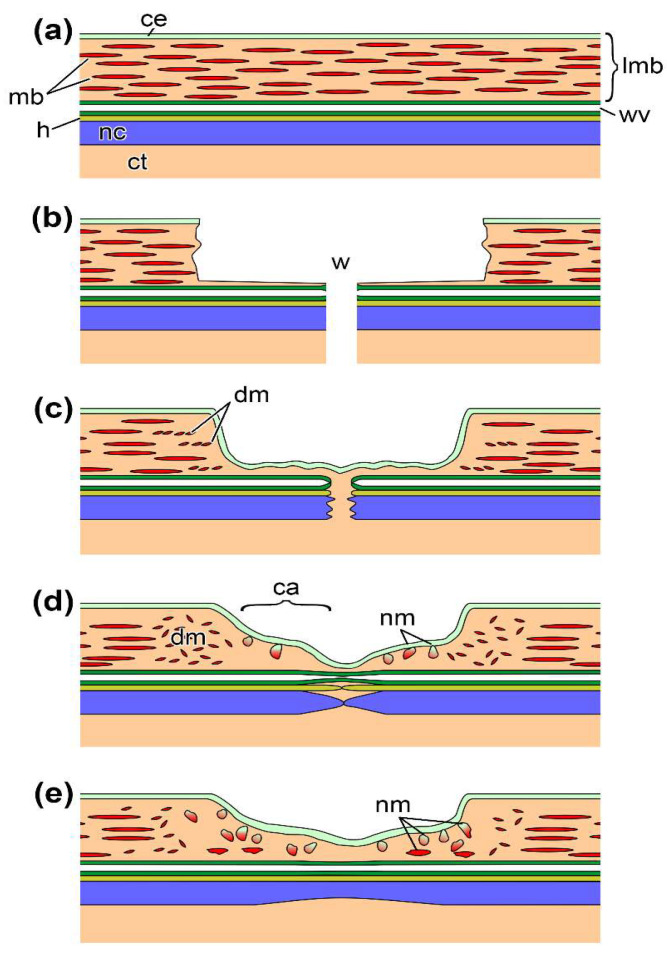
Scheme of a longitudinal section through ambulacrum at different stages after transverse cutting of a holothurian, *E. fraudatrix*. (**a**) Undamaged ambulacrum. (**b**) The ambulacrum immediately after cutting. (**c**) The ambulacrum at 2–4 dpd. (**d**) The ambulacrum at 10th dpd (first stage of regeneration). (**e**) The ambulacrum at 20th dpd (second stage of regeneration). ca, connective-tissue thickening (anlage); ce, coelomic epithelium; ct, connective tissue of the body wall; dm, destroyed muscle bundle; h, hemal lacuna; lmb, longitudinal muscle band; mb, muscle bundles; nc, radial nerve cord; nm, new muscle bundles; wv, radial water-vascular canal; w, wound.

Among the species with high regenerative capacities, representatives of the phylum Echinodermata are of particular interest. Many echinoderms can regenerate various organs, including muscles [9,10,11]. To date, the holothurian muscle system has been studied most thoroughly. These animals have large longitudinal muscle bands (LMBs) running along the radii of the body also referred to as ambulacra [12]. These are connective-tissue thickenings filled with muscle bundles [10]. Externally, LMBs are covered by coelomic epithelium. Each muscle bundle consists of 8–20 myocytes surrounded by the basal lamina (Figure 1) [13]. 

LMB is regenerated by coelomic epithelium cells [10,13,14] that undergo myogenic transformation, are embedded in the underlying connective tissue, and form muscle bundles. The LMB regeneration on the cellular level has been described in most detail from the holothurian *Eupentacta fraudatrix*. 

In this species, the LMB regeneration after damage takes 30–40 days [10]. The wound healing process induced by a transverse dissection of the body wall and ambulacrum lasts for 2–4 days. At 3–5 days post damage (dpd), cells of the coelomic epithelium of the LMB and interradii in the wound area begin to dedifferentiate and migrate to the damage site (Figure 1). The extracellular matrix (ECM) is formed at the ends of the muscle, where the connective-tissue anlagen emerge and grow toward each other within 7–10 dpd. Various proteinases such as, in particular, matrix metalloproteinases (MMPs) play an important role in their formation [15]. Inhibition of proteinases leads to a complete arrest of the LMB regeneration [16].

Simultaneously, the myogenic differentiation of coelomic epithelium cells begins. Small bundles of myofilaments are found in their cytoplasm. Groups of such myogenic cells are embedded in the ECM of muscle anlagen. While being embedded, myogenic cells differentiate into myocytes and form muscle bundles. At 15–20 dpd, the growing ends of the LMB get connected. However, the regeneration mechanism does not change: the formation of connective tissue, the myogenic transformation of coelomic epithelium cells, their embedding, and the muscle bundle formation continue. At 30–40 dpd, the integrity and size of the LMB at the damage site become fully restored. 

Currently, there is a lack of data on the molecular mechanisms of myogenesis of somatic muscles in echinoderms. Several studies have been published on the expression, localization, and functions of some genes associated with de- and redifferentiation of myoepithelial cells during the gut regeneration in the holothurian *Holothuria glaberrima* [17,18]. In this regard, we obtained and analyzed the transcriptome of regenerating LMBs from the holothurian *E. fraudatrix*. In the present study, we focused on two groups of genes playing a major role in regeneration: transcription factors (TFs) and MMPs.

## 2. Results and Discussion 

### 2.1. De Novo Transcriptome Assembly

**Figure 2:**
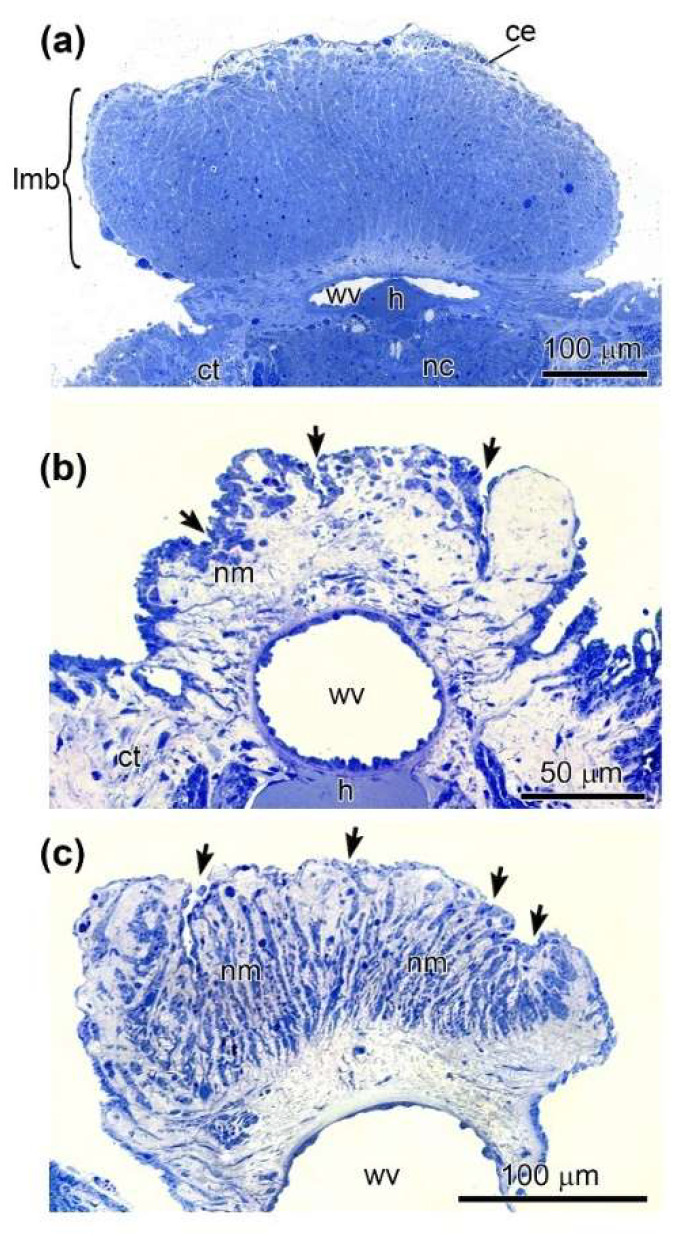
Transverse semithin sections of LMB at different stages after cutting of a holothurian, *E. fraudatrix*. (**a**) Undamaged LMB. (**b**) The LMB at 10th dpd (first stage of regeneration). (**c**) The LMB at 20th dpd (second stage of regeneration). ce, coelomic epithelium; ct, connective tissue of the body wall; h, hemal lacuna; lmb, longitudinal muscle band; nc, radial nerve cord; nm, new muscle bundles; wv, radial water-vascular canal; arrows indicate the sites of immersion of myogenic cells in the connective tissue.

To control the regeneration process, transverse semithin sections of intact muscle and LMB at two stages of regeneration were made. The intact LMB is filled with muscle bundles (Figure 2a). It is covered with a flattened coelomic epithelium. At 10th dpd, a connective tissue anlage was formed (Figure 2b). Cells of the coelomic epithelium begin to sink into it, forming new muscle bundles. At 20th dpd, active myogenesis is observed. Cells of the coelomic epithelium are immersed in the anlage in many places (Figure 2c). As a result, the connective tissue of the muscle is filled with new muscle bundles.

As a result of the sequencing of nine libraries corresponding to two stages of LMB regeneration and normal muscles from the holothurian *E. fraudatrix*, we obtained a total of 515 million (515,429,309) raw paired-end reads. After filtering and trimming of adapters, reads of good quality values made up 96%, with an average quality of 35.7 units by PhredScore 33 and an average read length of 99.2 nucleotides (see Supplementary Table S1). All of these reads, as well as the reads from the gut regeneration experiment (BioProject PRJNA509334), were used in the assembly. As a result of the assembly, filtering of contaminant and non-coding sequences, and subsequent clustering to identify isoforms, a total of 82,244 transcript isoforms and 66,001 genes were obtained. We assessed the completeness of the assembly using BUSCO v3 [19]. Thus, 98.4% of the core metazoan genes (based on 954 core essential genes) and 92.9% of the single-copy orthologs among them were identifiable in the transcriptome.

### 2.2. Differential Expression Analysis

**Figure 3:**
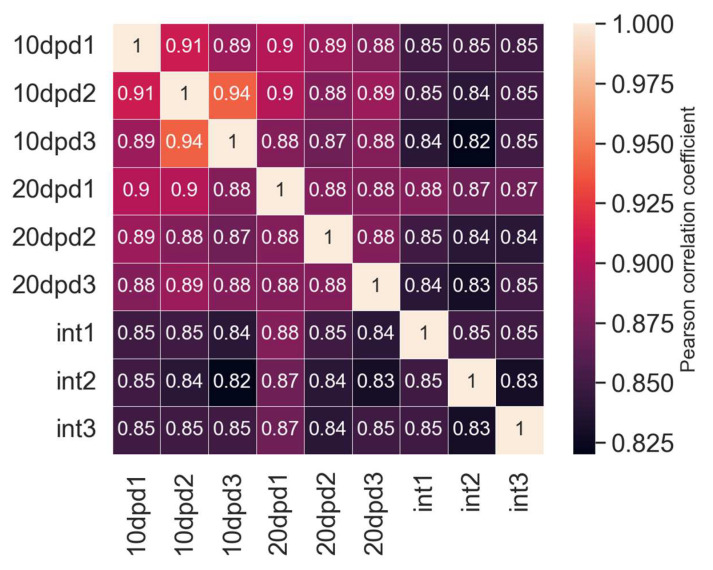
Correlation map of all RNA-seq samples and replicates. 10 dpd and 20 dpd—the first and second stages of regeneration, int—intact sample.

After aligning the reads to assembly and counting mapped events (see Supplementary Table S2), we performed a correlation analysis (Figure 3). We found a high correlation of replicates within the samples, at almost 88%, while the correlation of replicates in the samples from the regeneration stages was slightly higher (0.91 and 0.88) as compared to that in the sample from the intact LMB (0.85).

**Figure 4:**
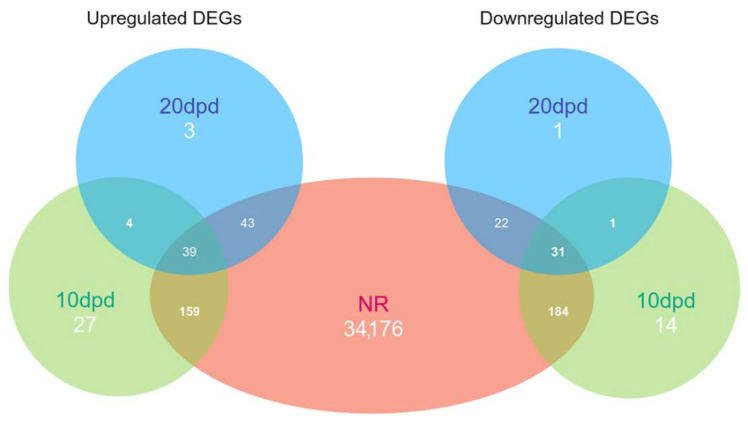
Venn diagram of up- and downregulated DEGs. NR—set of sequences with hits in the NCBI non-redundant database.

As a result of a search for differential expressed genes (DEGs), we found only 459 and 144 genes with noticeable changes in expression levels for 10 and 20 dpd samples, respectively, relative to the intact LMB (see Supplementary Tables S3 and S4). Of these, 413 and 135 sequences, respectively, had significant hits in the NRP NCBI database (Figure 4). Thus, most of the DEGs were detected when the two regeneration stages were compared with the intact sample. The same was evidenced by the map of correlations between the samples (Figure 3). The observation indicates essential changes in the work of genes after damage. This can probably provide identification of the regulatory genes whose expression is activated or repressed during regeneration.

### 2.3. Annotation

The annotation of 82,244 sequences by a BLASTx search against the NRP NCBI database resulted in the identification of 34,654 sequences and 25,784 genes with significant hits, including 5053 sequences having only unnamed hits (see Supplementary Table S5). The hits belonged to 1175 organisms, and 78.7% of the sequences matched echinoderm proteins (see Supplementary Table S6). In addition, there were about 3% of non-Metazoa sequences that might be contaminants. However, the lack of a genome for this or any related holothurian species did not allow us to accurately identify the source of these sequences. The annotation against human and sea urchin (*Strongylocentrotus purpuratus*) proteins led to the identification of 10,600 and 14,221 orthologs, respectively, as we used a modified reciprocal method to find the best orthologs (see Supplementary Tables S7 and S8).

### 2.4. Search for Genes of Transcription Factors with Differential Expression during LMB Regeneration

**Figure 5:**
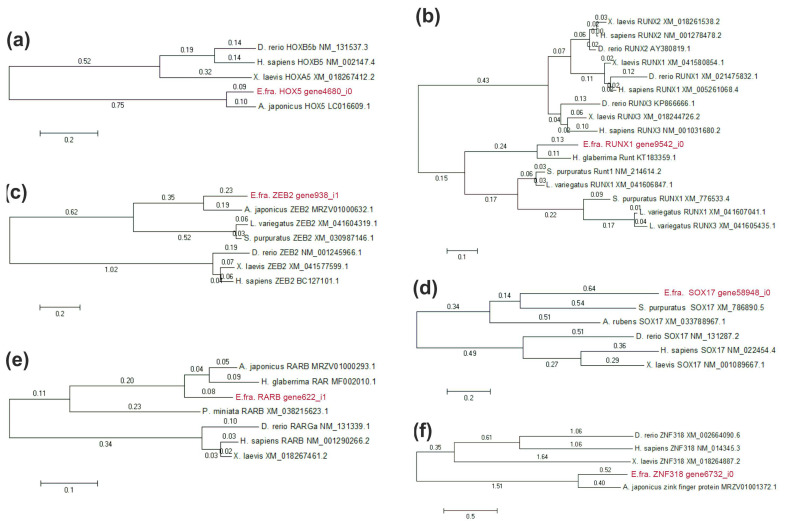
Phylogenetic trees showing the relationships of the TF sequences of the *E. fraudatrix* with homolog proteins of other animals. (**a**) HOX5; (**b**) RUNX1; (**c**) ZEB2; (**d**) SOX17; (**e**) RARB; (**f**) ZNF-318. The values on the branches indicate their length. The phylogenetic trees were constructed using the Maximum Likelihood algorithm in the MEGA11 program.

**Figure 6:**
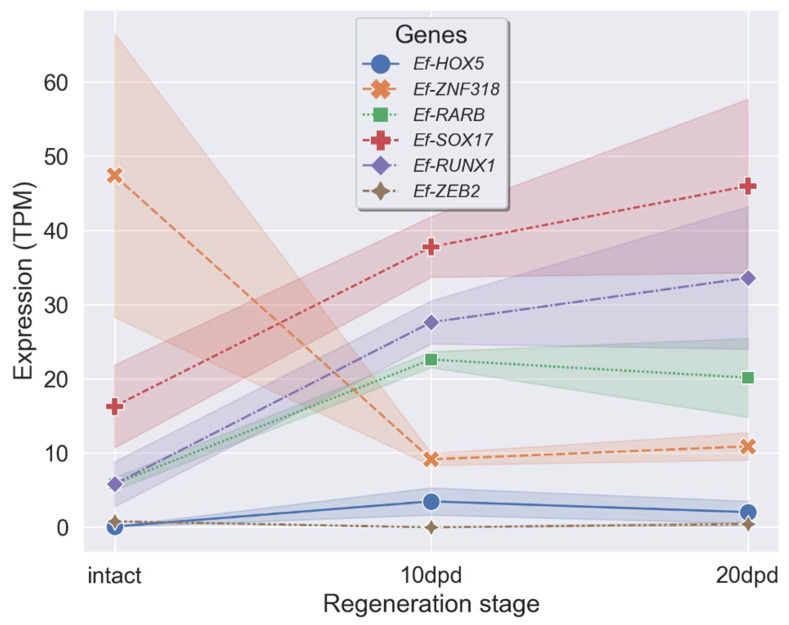
TPM values of the TF genes with significant changes in the expression level compared to the intact sample during the regeneration in *E. fraudatrix*.

A search for homologs of sea urchin and human TFs resulted in the identification of 174 and 926 TFs, respectively (see Supplementary Table S9). Of these, 117 TF homologs were found among proteins of both humans and sea urchins. Only seven of these TFs showed significant differential expression at one or both stages of regeneration. All homologs were verified using the NRP NCBI database and by constructing phylogenetic trees with orthologs of echinoderms and vertebrates (Figure 5). *Ef-ZFY* was removed because it had an unconditional match to known proteins. The expression profiles of the six remaining TFs, with TPM values at each stage, are shown in Figure 6. They belong to five TF classes: homeodomain factors (Ef-HOX5, Ef-ZEB2), nuclear receptors with C4 zinc fingers (Ef-RARB), runt domain factors (Ef-RUNX1), high-mobility group domain factors (Ef-SOX17), and C2H2 zinc finger factors (Ef-ZNF318).

*Ef-HOX5* belongs to homeotic (HOX) genes that are present in all bilateral animals, as well as in cnidarians [20,21]. The major function of HOX proteins is the sequential specification of body segments along the anteroposterior axis in ontogeny [22]. HOX are also involved in the regulation of numerous cellular processes such as apoptosis, proliferation, migration, and differentiation [23]. There is no convincing data on the involvement of *HOX* genes in the muscle regeneration, but these are known to be involved in the myogenesis during the embryonic development of vertebrates and insects [24]. *HOXb5*, the ortholog of *Ef-HOX5* in vertebrates, regulates the differentiation of angioblasts and mature endothelial cells from their mesodermal precursors, and also the differentiation of cell lineages of the vagus nerve and the neural crest [25,26]. Expression of a number of *HOX* genes increases during the gut regeneration in the holothurian *H. glaberrima* and the radial nerve regeneration in the sea star *Asterias rubens* [17,27]. The expression of *Ef-HOX5* increases during the LMB regeneration in *E. fraudatrix* (Figure 6). At 10 dpd, the number of its transcripts grows 38-fold; at 20 dpd, it grows 18-fold. The variation in the expression of *Ef-HOX5* indicates a possible involvement of Ef-HOX5 in the LMB regeneration. 

*Ef-ZEB2* belongs to the ZEB family that comprises two genes in vertebrates and one in echinoderms [28]. ZEB TFs trigger the epithelial–mesenchymal transition through the repression of epithelial genes, perform the function of maintaining the properties of stem cells, and prevent apoptosis [29,30]. These also take an active part in the development and regeneration of muscles. ZEB1 supports the resting state of myogenic precursors, preventing them from premature activation after injury, and is also involved in the differentiation of smooth muscle cells in the embryonic development of mice [31,32]. ZEB2 induces the myogenic differentiation of pluripotent stem cells and myosatellite cells [33]. In *E. fraudatrix*, a marked decrease in the number of *Ef-ZEB2* transcripts is observed at 10 dpd. This decrease may be explained by the need to prevent dedifferentiated cells of coelomic epithelium from premature myogenic transformation during their migration to the connective-tissue anlagen of LMB.

The ligand-dependent transcription regulator RARB is a component of the retinoic acid (RA) signaling pathway [34]. During the muscle regeneration in vertebrates, the RA signaling pathway regulates proliferation, differentiation, and apoptosis of fibroadipogenic precursors (FAP), non-muscle cells that, in turn, influence the myogenesis [35]. The involvement of the RA signaling pathway in regeneration is also reported for holothurians. Experiments with RAR inhibition during the gut regeneration in *H. glaberrima* have shown that this protein is involved in the muscle cell dedifferentiation [36]. In *E. fraudatrix*, the number of *Ef-RAR* transcripts at 10 and 20 dpd increases 4- and 3-fold, respectively, compared to the normal (control) group. It is likely that Ef-RAR during the LMB regeneration can also be involved in the dedifferentiation of myoepithelial cells of coelomic epithelium in the wound area.

RUNX TFs are common among all members of the Metazoa, playing an important role in oncogenesis, hematopoiesis, osteogenesis, proliferation, differentiation, and, possibly, dedifferentiation of cells [37,38,39,40,41,42,43]. In the sea urchin *S. purpuratus*, RUNX controls cell proliferation, survival, and differentiation at the embryonic and larval stages of development [44,45,46,47]. The increased expression of *Ef-RUNX1* during regeneration suggests its possible role in the myogenic differentiation of coelomic epithelium cells.

SOX17 belongs to the SOX family whose proteins are involved in various cellular processes in ontogeny [48]. During the vertebrate muscle regeneration, SOX17 affects self-renewal and inhibits differentiation of myosatellite cells [49]. In *E. fraudatrix*, *Ef-SOX17* is expressed in dedifferentiated cells of coelomic epithelium of the regenerating gut [50]. During the LMB regeneration, the number of *Ef-SOX17* transcripts increases markedly. We suggest that during the LMB regeneration, Ef-SOX17 may be involved in the dedifferentiation of coelomic epithelium cells or in maintaining their dedifferentiated state.

The *Ef-ZNF318* gene is an ortholog of *ZNF318* in vertebrates. At 10 dpd, the number of *Ef-ZNF318* transcripts decreases almost 5-fold compared to the normal (control) group. Then, at 20 dpd, the expression increases, with its value approaching those characteristics of intact LMB. There is a lack of data on the function of ZNF318 in developmental processes. The variation in the expression of *Ef-ZNF318* may indicate the involvement of Ef-ZNF318 in the LMB regeneration in *E. fraudatrix*.

### 2.5. Search for Genes of MMPs and ECM Proteins with Differential Expression during LMB Regeneration

A search for *MMP* homologs of sea urchin (*S. purpuratus*) and human in *E. fraudatrix* revealed 20 and 17 MMPs, respectively (see Supplementary Table S10). A total of 15 MMP homologs were found among both sea urchin and human proteins. Of these, only seven showed significant variations in expression at one or both stages of LMB regeneration (see Supplementary Table S10). All the homologs with differential expression were verified using the Echinobase database and by constructing a phylogenetic tree with MMPs of the sea urchins *S. purpuratus* and *H. sapiens* (Supplementary Figure S1). The sequences were identified by both methods as *Ef-MMP11*, *Ef-MMP13*, *Ef-MMP13-1*, *Ef-MMP16-2*, *Ef-MMP16-3*, *Ef-MMP24*, and *Ef-MMP24-1*. 

**Figure 7:**
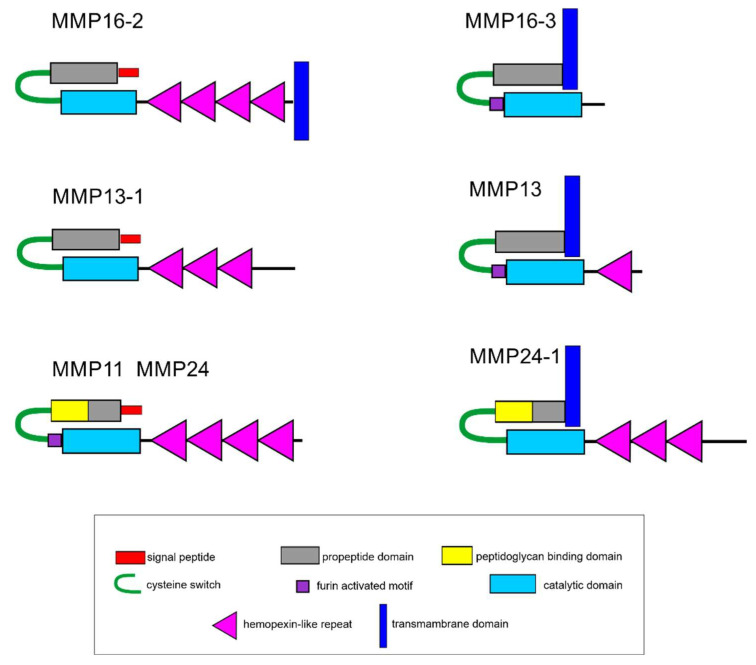
Scheme of structure of the differential expressed matrix metalloproteinases in *E. fraudatrix*.

In its domain structure, Ef-MMP13-1 belongs to archetypal MMPs, while Ef-MMP11, Ef-MMP13, Ef-MMP16-3, Ef-MMP24, and Ef-MMP24-1 belong to furin-activatable MMPs (Figure 7). The Ef-MMP16-2 proteinase lacks furin-activated motif but has a transmembrane domain at C-terminus, which is not typical of archetypal MMPs. Proteinases with a similar structure are characteristic of echinoderms [51]. Out of the seven identified DEGs of MMPs, the expression decreases during regeneration only in *Ef-MMP13-1*.

The MMP subfamily comprises 24 genes in humans and 17–23 genes in echinoderms [51,52]. Their major function consists of remodeling the ECM [53,54]. In case of damage to vertebrate muscle, MMPs prevent tissue fibrosis due to their ability to break down collagen and, thereby, make the tissue more accessible to migrating myogenic cells [54,55,56]. 

In echinoderms, including *E. fraudatrix*, MMPs also play an important role in the processes of development, regeneration, and asexual reproduction [50,51,57]. In the latter species, transcripts of the *MMP* genes and the tissue inhibitor of metalloproteinases are found in the gut anlage during the digestive system regeneration [50]. Apparently, various MMPs perform different functions in the gut formation. When proteases are blocked by the GM6001 inhibitor, the LMB regeneration in *E. fraudatrix* is completely arrested [16]. Thus, the variation in the expression of *MMP* genes during the LMB regeneration in *E. fraudatrix* indicates their possible involvement in this process.

Connective tissue plays an important role in the regeneration of LMBs [10,13]. A search for genes of ECM proteins with differential expression revealed 19 genes (see Supplementary Table S11). Of these, only *short-chain C4-like collagen* and *fibrillin-2* are overexpressed during ambulacrum regeneration. Short-chain collagen C4 is a component of basal membranes [57]. The increase in its expression is understandable, since the reorganization of epithelia of many organs occurs during the regeneration of ambulacrum (nerve cord, water-vascular canal, coelomic epithelium of interradii). Fibrillins are one of the main components of the connective tissue of echinoderms [57]. An increase in *fibrillin-2* expression during LMB regeneration indicates the participation of fibrillins in the formation of the ECM of LMB anlagen. Interestingly, the number of transcripts of collagen genes does not increase during ambulacrum regeneration. Perhaps the basis of muscle anlagen is fibrillin, and collagen begins to accumulate only after the formation of the main number of muscle bundles (30–40 dpd [16]). 

### 2.6. Network of Enrichment Biological Processes and Pathways

**Figure 8:**
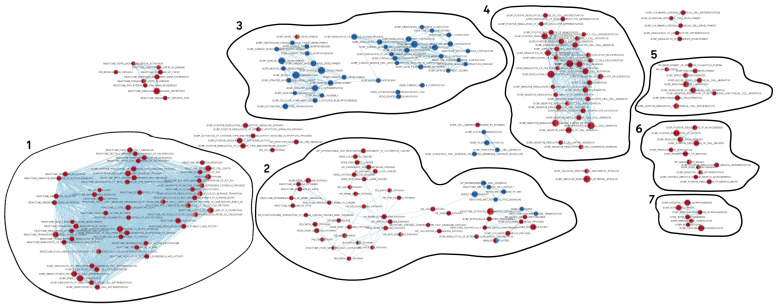
Network of enrichment biological processes and pathways during ambulacrum regeneration in *E. fraudatrix*. Nodes represent biological process (gene set). Edges represent overlap between pair of gene sets. Node size and edge width depend on the number of genes. Node fill represents enrichment scores of terms at the first (right half) and second (left half) stage of regeneration relative to the intact sample. The color gradient represents an increase (red) or decrease (blue) in the level of expression (depending on the enrichment score) in the regeneration, relative to the intact tissue. 1–7—blocks associated with the regeneration of various structures of ambulacrum. The full version of the network is provided in Supplementary Figure S2.

By constructing a network, we obtained a total of 570 enrichment pathways and biological processes (terms) combining 5199 homologs of human proteins (Figure 8). Of them, 482 and 73 terms had up- and downregulated genes, respectively, at both stages of regeneration compared to intact tissue.

As mentioned earlier [58], we cannot interpret the processes represented in the network literally, since the processes described for humans and other mammals constitute the basis of gene ontology. This particularly applies to the interpretation of groups of terms such as “biological process” and “molecular function”. Invertebrates may have a completely different set of organs than mammals, while the possibly homologous organs may have different structure and functions. Moreover, the greater the evolutionary distance between species, the more differences arise in the number of genes and, more importantly, in the composition of gene families. For this reason, it often becomes impossible to determine the orthology of many genes between animal species. The main body of information about genes’ functions is based on those of human genes and, therefore, the function of human gene homologs in invertebrates cannot be accurately determined by such an analysis. The example of the *SOX17* gene is quite illustrative. The structure of muscles and the mechanisms of their renewal differ rather substantially between echinoderms and vertebrates [8]. This probably explains the absence of the *Ef-SOX17* gene from the nodes associated with myogenesis in *E. fraudatrix*.

Nevertheless, the set of the network blocks is quite well consistent with the processes occurring during the ambulacrum regeneration. In addition to LMB, its other structures, such as the water-vascular canal, radial nerve cord, and body wall, were also damaged. The blocks associated with the regeneration of these structures can be seen in the reconstructed network. 

The network consists of seven blocks of closely interrelated processes associated with protein and RNA processing; cell differentiation and mitosis; extracellular matrix organization and bone morphogenesis; immune response and immune cell differentiation; neurogenesis; and muscle development. Since a major part of the network is occupied by the housekeeping processes, we deleted them (see Supplementary Figure S2). Almost all processes were significantly upregulated. However, it is worth noting that all the processes associated with the muscle organization and development were downregulated.

The organism’s first response to damage is immune system activation, which is also typical of echinoderms [59]. Blocks 1 and 4, having the largest number of nodes and internal links, contain processes related to various aspects of the immune system functioning (Figure 8). These probably reflect the activation of the immune system and the regeneration of the coelomocyte population. In the case of body wall damage, holothurians lose the coelomic fluid along with coelomocytes. The presence of GO terms, associated with the hematopoietic cell differentiation, cell cycle and mitotic activity, and various signaling pathways, in blocks 1 and 4 indicates the involvement of the genes of these blocks in the immune response regulation and the immune cell population recovery.

Block 5 contains GO terms related to migration and proliferation of endothelial cells (Figure 8). These processes probably correspond to the transformation of epithelia at the site of ambulacrum damage: the luminal epithelium of the water-vascular canal and coelomic epithelium on the LMB surface. The regeneration of the radial nerve cord, another ambulacral structure, is reflected in block 6 (Figure 8). Here, the GO terms associated with neurogenesis are brought together.

Interpreting block 2 is quite a challenge (Figure 8). It represents a large group of processes related to carcinogenesis. To date, researchers have not managed to detect or induce neoplasms in any organ of echinoderms. Since part of the GO terms is related to the activity of MMPs and the modification of connective-tissue proteins, this block is likely to reflect the transformation of ECM and its cells. Block 7 is also associated with the connective tissue transformation (Figure 8). It contains such GO terms as “skeletal system morphogenesis”, “bone morphogenesis”, etc. Obviously, these two blocks contain processes related to both skin wound repair and LMB regeneration. The ECM synthesis is required in both cases. In particular, muscle regeneration begins with the formation of a connective-tissue anlagen where coelomic epithelium cells are then embedded [13]. In this regard, the ECM synthesis and transformation play a major role in this process. Furthermore, it has been shown that the blocking of MMP leads to a complete arrest of the LMB regeneration in *E. fraudatrix* [16]. 

Block 3 reflects the process of muscle bundle formation (Figure 8). It contains GO terms related with the morphogenesis of the contractile system structures. Most of the processes it contains are downregulated. The only exception is where the process of heart valve development is activated. This can probably be explained by the high level of muscle tissue renewal in undamaged holothurians. In case of damage, these processes either slow down or stop. As a result, the expression of most genes during regeneration decreases compared to normal individuals. In addition, such a result may indicate a sharp difference in myogenesis between echinoderms and mammals.

In this regard, the node containing genes united by the GO term “heart valve development” is of particular interest. It is the only node in block 3 that contains upregulated genes (Figure 8). The presence of this GO term in the network of enrichment biological processes of regenerating ambulacrum in holothurians may be explained by some similarity of the morphological features of the heart valve formation in vertebrates with the LMB regeneration in echinoderms [60,61]. In both cases, the ECM is accumulated, and cells migrate into it. 

Among the identified TFs, ZNF318 and ZEB2 were not associated with any of the processes. This is probably due to insufficient information about the functions of these proteins. The greatest number of processes in the network is associated with RUNX1. These are processes such as immune cell differentiation, regulation of adhesion, and hematopoietic stem cell differentiation. HOXB5 is included in the bone morphogenesis, and RARB is involved both in the bone development and in the muscle and epithelial cell differentiation. SOX17 is only included in a separate node, not related to other blocks. This node contains the process of endoderm development, which is very puzzling, since there are no cells of endodermal origin in ambulacrum. This, once again, shows the challenge of interpreting GO for non-model species that are evolutionary remote from mammals.

Six of the seven identified DEGs of MMPs are included in block 2, in the nodes containing the processes of ECM remodeling. MMP13 is absent from the network, since this contig is not identified by the human database. Ef-MMP24 is probably the most multifunctional protease. It is present in many blocks, including block 3 associated with myogenesis.

## 3. Materials and Methods

### 3.1. Animals

Adult individuals of the holothurian *Eupentacta fraudatrix* (D’yakonov et al., 1958) were collected in Peter the Great Bay, Sea of Japan, and kept in 3 m^3^ tanks with running aerated seawater at 16 °C for one week. Transverse dissection of the right dorsal ambulacrum was made with scissors. As a result of the dissection, a nerve cord, a hemal vessel, a water-vascular canal, and a LMB were cut. Then, holothurians were placed into the tanks, where they regenerated their LMB and body wall.

### 3.2. Light Microscopy

For the microscopy analysis, a part of the body wall with undamaged or regenerating ambulacrum was sampled. A 2.5% glutaraldehyde solution prepared on 0.05 M cacodylate buffer (pH 7.4) was used as a fixative. The samples were fixed for 1–2 h at 4 °C. Then, the material was washed in 0.05 M cacodylate buffer (pH 7.4) and post-fixed for 1 h with 1% solution of OsO_4_, prepared on the same buffer. After that, samples were dehydrated with rising concentrations of ethanol followed by acetone, and embedded into a mixture of araldite M and Epon 812 (Fluka) according to a standard procedure. Semithin sections (0.7 μm) were made using Reichert Ultracut E ultramicrotome and stained with 1% methylene blue in a 1% water solution of sodium tetraborate. The analysis of semithin sections was carried out using a Leica DM 4500 B light microscope equipped with a Leica DFC 300 FX digital camera.

### 3.3. Sample Collection and RNA Extraction

Tissues were sampled from the normal individuals and the dissected ones on days 10 (first stage of regeneration) and 20 (second stage) post-damage. A total of nine individuals were selected from each of the stages. Each sample was represented in three biological replicates with three individuals, pooled together, per replicate. A small piece of ambulacrum was cut out from the epidermis and body wall and taken for analysis. 

Before isolating total RNA, the tissue sample was precipitated in sterile seawater. Homogenization was carried out in the ExtractRNA solution (Evrogen, Moscow, Russia) with metal balls on a TissueLyser LT homogenizer (Qiagen, Hilden, Germany). Total RNA was isolated by phenol–chloroform extraction [62]. RNA quality was checked at the Bio-Rad Experion station and only RNA samples with a RQI value greater than 8 units were accepted (see Supplementary Data S1–S3). Poly-A RNA was purified with Dynabeads Oligo (dT)25 magnetic beads (Thermo Fisher Scientific, Waltham, MA, USA).

### 3.4. Transcriptome Sequencing

The libraries were prepared using a NEBNext Ultra II Directional RNA Library Prep Kit (NEB #7760), and fragments with a length of 300–500 nucleotides, including adapters, were selected. After testing the quality on an Agilent TapeStation 4200, paired-end sequencing (2 × 100) was performed on an Illumina NovaSeq 6000 sequencing system. Raw reads were loaded to the SRA NCBI database with the accession nos. from SRR16928147 to SRR16928155 for normal muscle and for two stages of regeneration, respectively.

### 3.5. De Novo Transcriptome Assembly

Raw reads from nine libraries (listed in Supplementary Table S1) that we obtained in FASTQ format were processed using the Trimmomatic 0.39 software with the “LEADING:20 TRAILING:20 SLIDINGWINDOW:5:20 AVGQUAL:25 MINLEN:25” parameters to achieve clean reads by removing those containing adapter sequences, poly-N sequences, or low-quality bases [63].

Then, the clean reads were classified using the Kraken 2.1.2 software with a custom database that contained archeal, bacterial, viral, plasmid, human, UniVec Core, protozoan, and fungal sequences, and also rRNA from SILVA v138.1 and holothurian rRNA from NCBI (the sequences were downloaded on 23 June 2021). The confidence threshold value was obtained using a series of values from 0.0 to 0.8 and by plotting count of reads per unique taxonomy rank. All unclassified paired reads were assembled using the SPAdes 3.15.1 software with a k-mer length of 33 and 49 [64]. Subsequently, the obtained sequences were processed and assembled with the HomoloCAP script as described in [65]. The resulting sequences were filtered according to the NCBI requirements and uploaded to the TSA NCBI Database with the index GHCL00000000. Transcriptome completeness was assessed using BUSCO 5.2.1 [19] in the “protein” mode with a Metazoa 10 dataset.

### 3.6. Differential Expression Analysis

To find DEGs, the number of mapped reads was calculated in the RSEM 1.3.1 software [66]; paired-end reads were aligned in the Bowtie 2.4.4 software [67] with the following parameters: “--nofw --no-mixed --no-discordant --no-contain --gbar 1000 -N 1 --end-to-end -k 20 -q --maxins 1000”. After filtering out genes with a CPM value less than 0.5 in at least two replicates, differential expression was evaluated for the sequences in the DESeq2 1.28.1 software [68]. The control sample was an intact LMB. The DEGs with an expression level in the sample twice as high as that in the control and with the adjusted *p*-value less than 0.05 were considered actual.

### 3.7. Annotation

Annotation was carried out using several protein databases with a standard e-value for BLASTp 2.12.0 of 1 × 10^−5^. Basic annotation was carried out using the NR NCBI Database (19.12.2021); the annotation for enrichment analysis was performed by BLASTp search against human proteins from the Ensemble database v105 [69]; the annotation for finding TFs was based on human proteins from the Ensemble database and sea urchin proteins from the Echinobase project [70]. Orthologs of human proteins were identified using a custom Python script that implements modified reciprocal method for finding the best hit, as described in the article [58]. The list of human TFs was taken from The Human Transcription Factors database v1.01. The list of sea urchin TFs was taken from Echinobase (2.12.2021).

The enrichment analysis of biological processes and pathways was performed in the GSEA 4.1 software [71] in accordance with the EnrichmentMap protocol for RNA-seq data [72]. The gene sets for biological processes and pathways were accessed from the MsigDB database v7.4 [71]. Then, the results of the enrichment analysis were visualized using the EnrichmentMap plug-ins in the Cytoscape 3.9 software [73] (see Supplementary Figure S2).

### 3.8. Phylogenetic Tree Construction

To construct phylogenetic trees, we used putative amino acid sequences and conserved regions of putative amino acid sequences of the holothurians *E. fraudatrix*, *Apostichopus japonicus* (Selenka, 1867), and *Holothuria glaberrima* (Risso, 1826), the sea urchins *Strongylocentrotus purpuratus* (Stimpson, 1857) and *Lytechinus variegatus* (Lamarck, 1816), the sea stars *Asterias rubens* (Linnaeus, 1758) and *Patiria miniata* (Brandt, 1835), and also the vertebrates *Homo sapiens* (Linnaeus, 1758), *Danio rerio* (Hamilton, 1822), and *Xenopus laevis* (Daudin, 1802). Determination of conserved regions of the putative amino acid sequences was carried out using the Gblock program [74].

The phylogenetic trees were constructed by the Maximum Likelihood method using the Mega v11 [75] and MrBayes 3.2 [76] software. For constructing a MrBayes tree, conserved regions of putative amino acid sequences were analyzed in Partitionfinder 2.1.1 [77]. Trees were visualized using the iTOL v6.6 online tool (https://itol.embl.de; accessed on October 2022). 

### 3.9. MMP Domain Structure

The domain structure was determined using the Pfam (http://pfam.xfam.org, accessed on accessed on 20 September 2022), Blast NCBI, and Smart (http://smart.embl-heidelberg.de, accessed on 20 September 2022) programs. In addition, SignalP-5.0 Server (http://www.cbs.dtu.dk/services/SignalP, accessed on 20 September 2022) and Phobius (https://phobius.sbc.su.se, accessed on 20 September 2022) were used to more accurately detect the presence of a signal peptide and transmembrane domains in a protein molecule.

## 4. Conclusions

Our study has shown that expressions of many genes, including TFs and MMPs, vary during the ambulacrum regeneration in *E. fraudatrix*. Some of them are obviously related to the regulation of wound healing and the regeneration of the nerve cord and water-vascular canal. Among the genes that can be involved in the LMB regeneration, six DEGs of TFs and seven DEGs of MMPs can be distinguished. Four TF genes have an increased expression and two have a decreased expression compared to those in normal holothurians. Out of the seven MMP genes, six showed an increase in the number of transcripts during the regeneration, and one showed a decrease.

The analysis of sequencing data has confirmed the morphological data on the presence of two processes in the LMB regeneration: ECM transformation and myogenesis. In this regard, of particular interest is the presence of upregulated genes of the GO term “heart valve development” in the network of enrichment biological processes. The similarity between the LMB formation in holothurians and the heart valve formation in vertebrates on the morphological and molecular levels may indicate the antiquity of this mechanism of mesodermal structure transformation, which was co-opted into various morphogeneses in different deuterostome groups.

## Data Availability

The raw reads that we obtained have been uploaded to the SRA NCBI Database with accession nos. from SRR16928147 to SRR16928155. The assembly has been uploaded to the TSA NCBI Database with the index GHCL00000000.

## Supplementary Materials

The following supporting information can be downloaded at: https://www.mdpi.com/article/10.3390/ijms232416037/s1.
