# Supplementary material for: Muscle Regeneration in Holothurians without the Upregulation of Muscle Genes
Source: Int J Mol Sci. 2022 Dec 16;23(24):16037. doi: 10.3390/ijms232416037 (PMC9785333; doi:10.3390/ijms232416037)
Supplement: Supplementary file 1 [file ijms-23-16037-s001.zip › Data/Data S1.pdf]

Run Overview Report

**Project:**	efra.muscle.rna	**Acq. Analyst:**	DefaultUser
**Assay:**	Eukaryote Total RNA StdSens	**Acq. Time:**	5/8/2021 8:32:00 PM
**Run:**	int-1.2.3rep_20-1.2rep_5-8-2021_8-31-59 PM	**Signature:**	N/A
**Run Version:**	N/A		

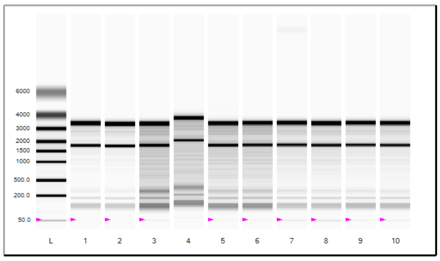

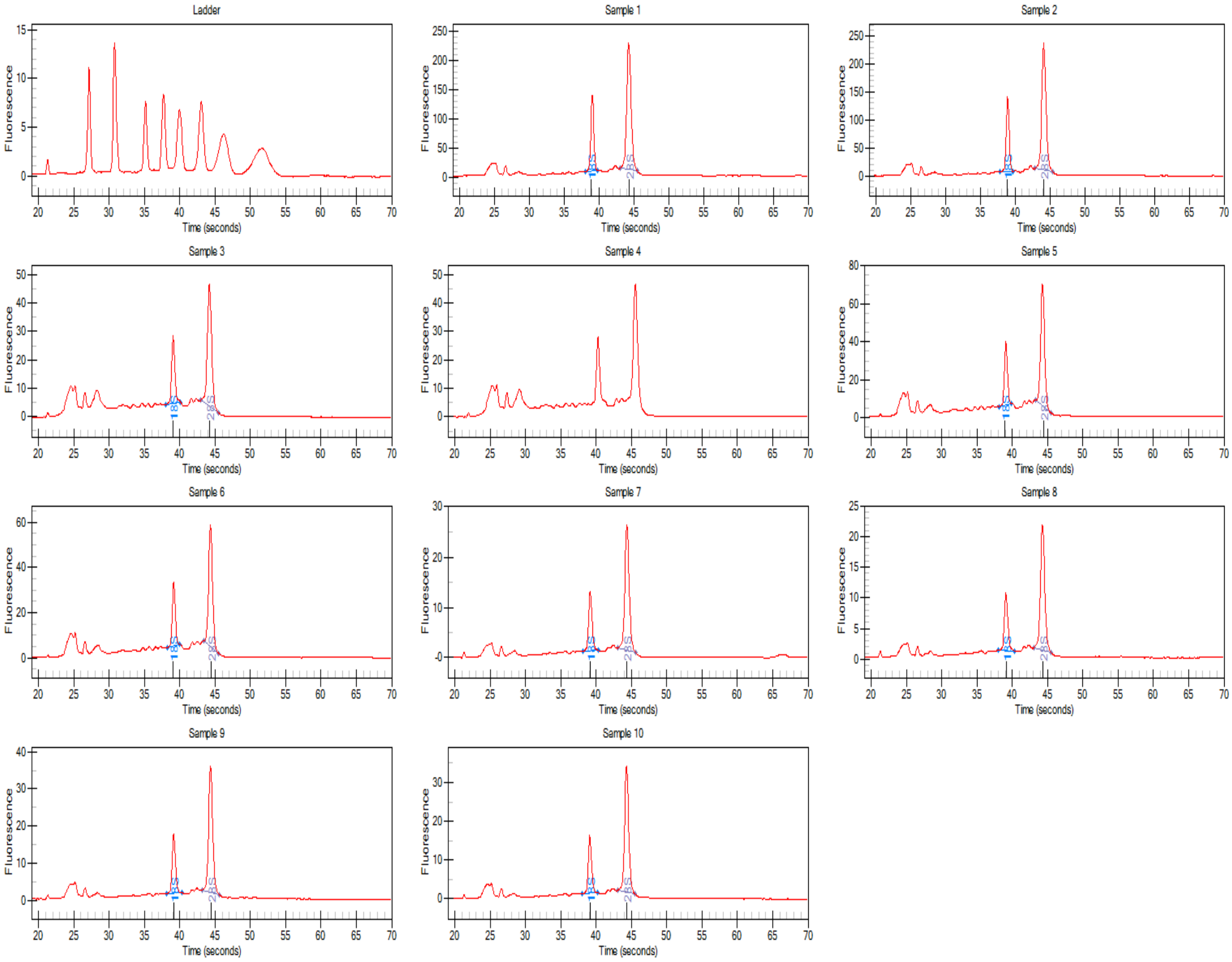

Project: efra.muscle.rna
Assay: Eukaryote Total RNA StdSens
Run: int-1.2.3rep_20-1.2rep_5-8-2021_8-31-59 PM
Run Version: N/A

Acq. Analyst: DefaultUser
Acq. Time: 5/8/2021 8:32:00 PM
Signature: N/A

Well# Ladder

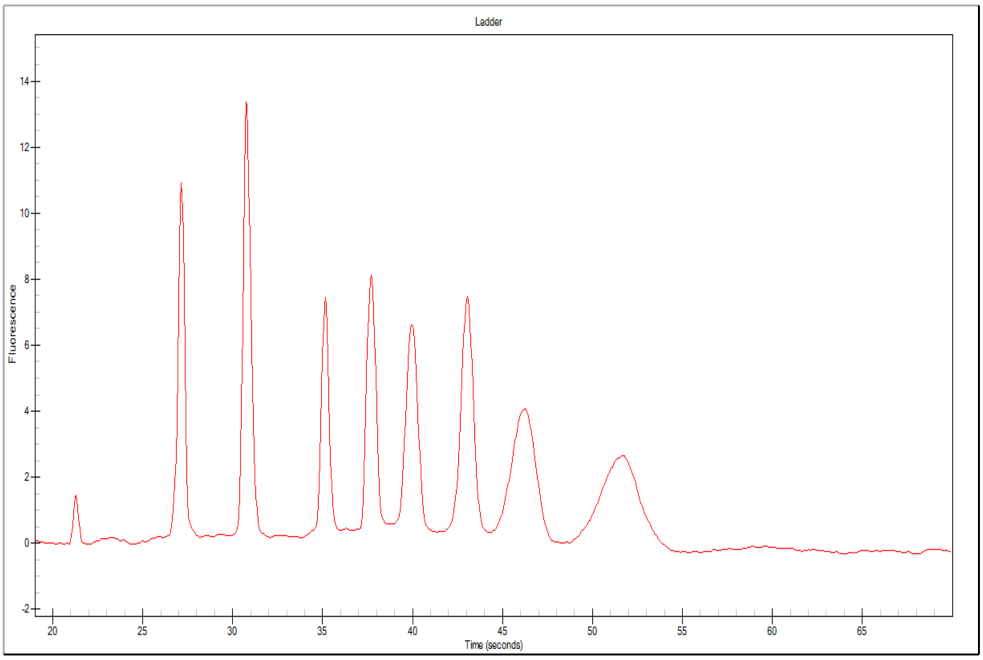

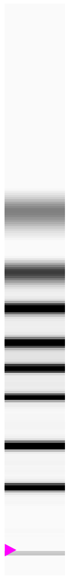

L

Well# Ladder

RNA Area: 134.22
RNA Concentration: 160.00 ng/µl

Well# Ladder

Peak State	Peak Number	Mig. Time (secs)	Corrected Area	Comments
	1	21.30	2.24	
L	2	27.15	17.59	
L	3	30.75	21.89	
L	4	35.15	10.82	
L	5	37.70	13.54	
L	6	39.95	13.97	
L	7	43.05	15.12	
L	8	46.25	14.93	
	9	51.70	14.34	

Project: efra.muscle.rna
Assay: Eukaryote Total RNA StdSens
Run: int-1.2.3rep_20-1.2rep_5-8-2021_8-31-59 PM
Run Version: N/A
Acq. Analyst: DefaultUser
Acq. Time: 5/8/2021 8:32:00 PM
Signature: N/A

Well# 1 Sample 1

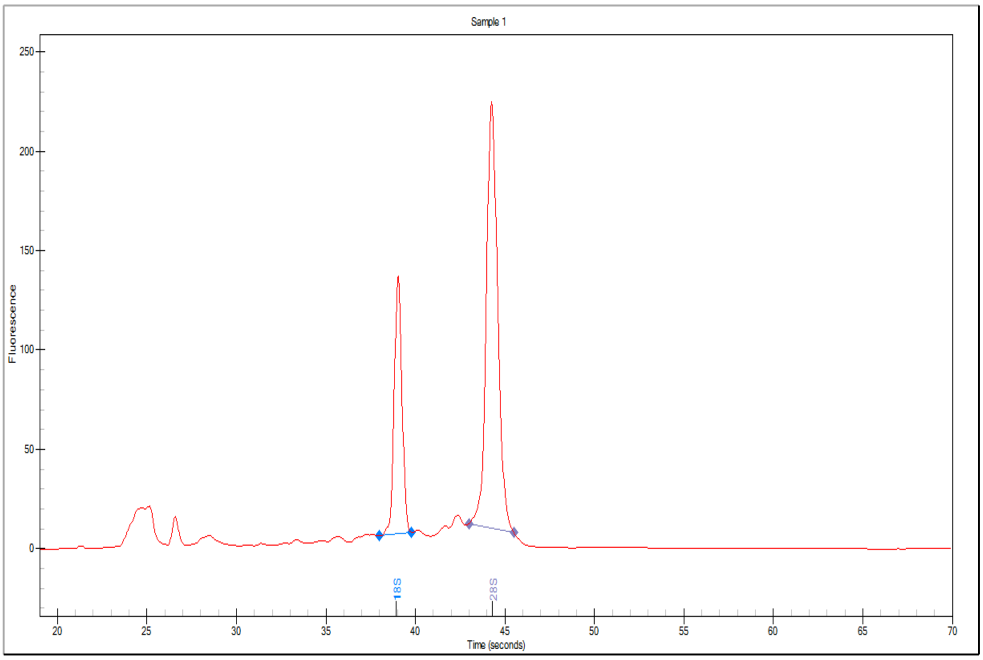

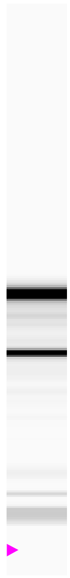

Well# 1 Sample 1					
Fragment Number	Fragment Name	Start Time	End Time	Area	% of Total Area
1	18S	38.00	39.80	168.54	17.01
2	28S	43.05	45.55	355.86	35.93

RNA Area: 990.54
RNA Concentration: 1,180.83 ng/µl
Ratio[28S/18S]: 2.11

RQI: 9.9

Well# 1 Sample 1				
Peak State	Peak Number	Mig. Time (secs)	Corrected Area	Comments
	1	21.30	1.83	
	2	24.78	74.23	
	3	25.12	44.80	
	4	26.56	26.50	
	5	28.50	28.44	
	6	30.58	2.88	
	7	31.38	4.16	

Egram, Gel Lane and Result Table Report

Project: efra.muscle.rna
Assay: Eukaryote Total RNA StdSens
Run: int-1.2.3rep_20-1.2rep_5-8-2021_8-31-59 PM
Run Version: N/A
Acq. Analyst: DefaultUser
Acq. Time: 5/8/2021 8:32:00 PM
Signature: N/A

Well# 1 Sample 1

Peak State	Peak Number	Mig. Time (secs)	Corrected Area	Comments
	8	32.67	3.71	
	9	33.37	8.41	
	10	34.76	8.24	
	11	35.65	13.34	
	12	39.03	198.12	
	13	40.12	18.52	
	14	41.66	20.19	
	15	42.35	29.33	
	16	44.24	420.59	

Egram, Gel Lane and Result Table Report

Project: efra.muscle.rna
Assay: Eukaryote Total RNA StdSens
Run: int-1.2.3rep_20-1.2rep_5-8-2021_8-31-59 PM
Run Version: N/A

Acq. Analyst: DefaultUser
Acq. Time: 5/8/2021 8:32:00 PM
Signature: N/A

Well# 2 Sample 2

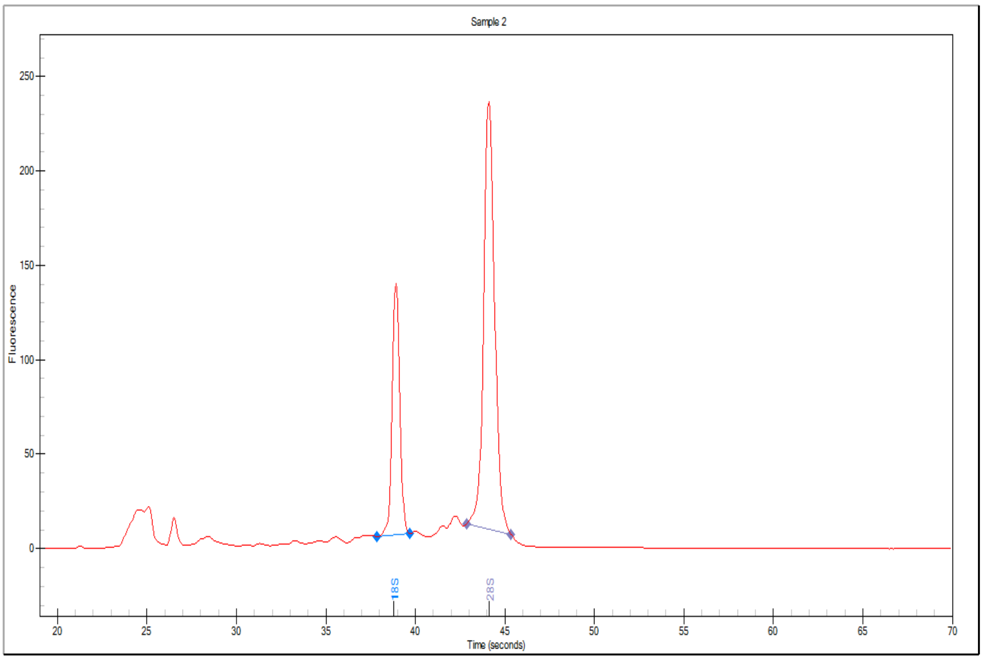

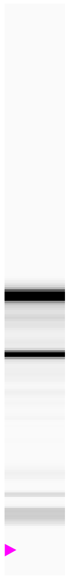

2

**Well# 2 Sample 2**					
Fragment Number	Fragment Name	Start Time	End Time	Area	% of Total Area
1	18S	37.90	39.70	169.61	16.96
2	28S	42.90	45.35	362.89	36.28

RNA Area: 1,000.16
RNA Concentration: 1,192.30 ng/µl
Ratio[28S/18S]: 2.14

RQI: 9.9

**Well# 2 Sample 2**				
**Peak State**	**Peak Number**	**Mig. Time (secs)**	**Corrected Area**	**Comments**
	1	21.30	1.69	
	2	24.60	73.69	
	3	25.09	47.48	
	4	26.51	26.27	
	5	28.38	27.83	
	6	30.55	2.84	
	7	31.29	4.24	

Project: efra.muscle.rna
Assay: Eukaryote Total RNA StdSens
Run: int-1.2.3rep_20-1.2rep_5-8-2021_8-31-59 PM
Run Version: N/A

Acq. Analyst: DefaultUser
Acq. Time: 5/8/2021 8:32:00 PM
Signature: N/A

Well# 2 Sample 2

Peak State	Peak Number	Mig. Time (secs)	Corrected Area	Comments
	8	32.56	3.49	
	9	33.25	7.86	
	10	34.68	8.42	
	11	35.57	13.01	
	12	38.91	198.37	
	13	39.99	17.95	
	14	41.52	20.04	
	15	42.21	29.82	
	16	44.08	426.32	

Egram, Gel Lane and Result Table Report

**Project:**	efra.muscle.rna	**Acq. Analyst:**	DefaultUser
**Assay:**	Eukaryote Total RNA StdSens	**Acq. Time:**	5/8/2021 8:32:00 PM
**Run:**	int-1.2.3rep_20-1.2rep_5-8-2021_8-31-59 PM	**Signature:**	N/A
**Run Version:**	N/A		

Well# 7 Sample 7

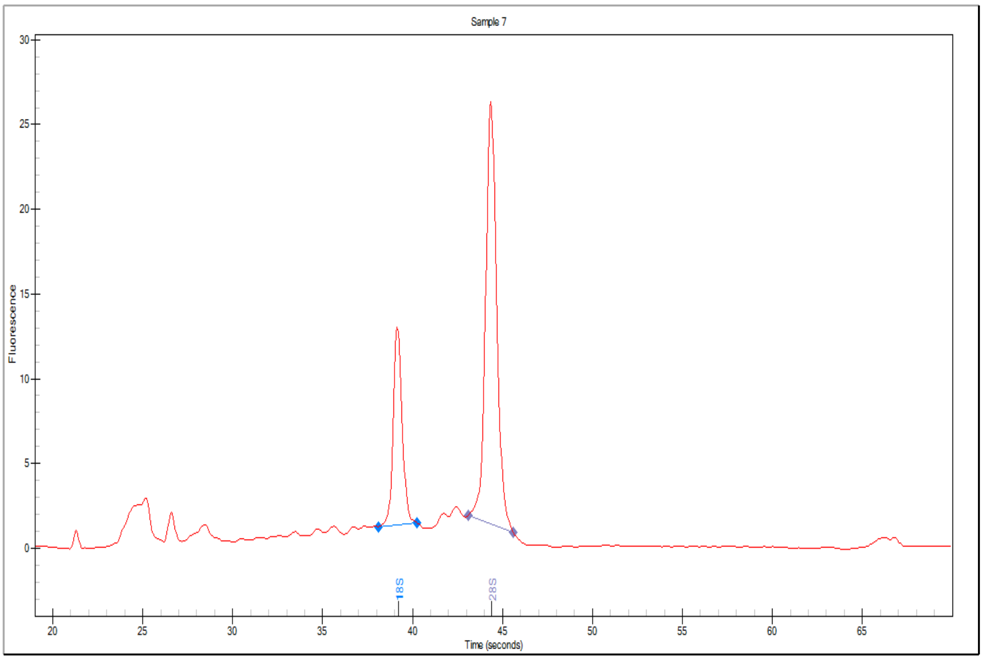

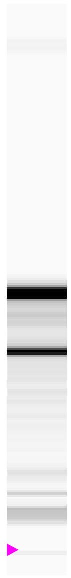

7

Well# 7 Sample 7

Fragment Number	Fragment Name	Start Time	End Time	Area	% of Total Area
1	18S	38.10	40.30	17.26	13.41
2	28S	43.15	45.60	40.09	31.16

RNA Area: 128.65
RNA Concentration: 153.36 ng/µl
Ratio[28S/18S]: 2.32

RQI: 9.0

Well# 7 Sample 7

Peak State	Peak Number	Mig. Time (secs)	Corrected Area	Comments
	1	21.30	1.42	
	2	25.20	4.92	
	3	26.60	2.48	
	4	28.43	1.73	
	5	39.13	17.75	
	6	42.41	2.54	
	7	44.33	45.72	

Egram, Gel Lane and Result Table Report

Project: efra.muscle.rna
Assay: Eukaryote Total RNA StdSens
Run: int-1.2.3rep_20-1.2rep_5-8-2021_8-31-59 PM
Run Version: N/A
Acq. Analyst: DefaultUser
Acq. Time: 5/8/2021 8:32:00 PM
Signature: N/A

Well# 8 Sample 8

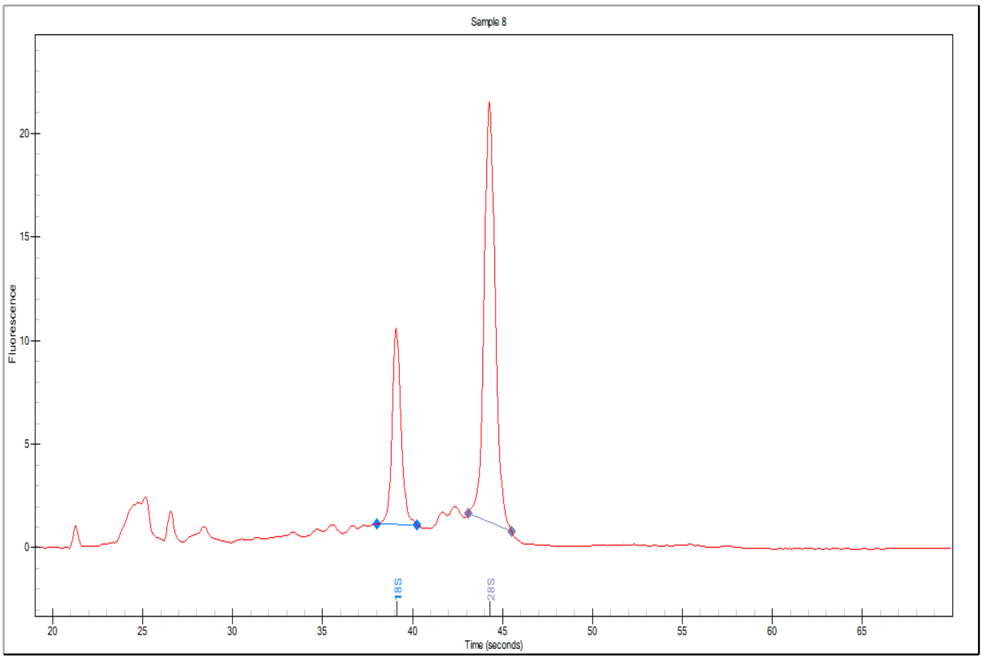

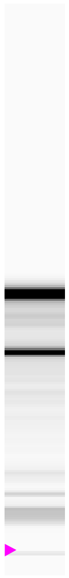

8

Well# 8 Sample 8

Fragment Number	Fragment Name	Start Time	End Time	Area	% of Total Area
1	18S	38.00	40.25	14.01	12.58
2	28S	43.10	45.50	32.83	29.48

RNA Area: 111.34
RNA Concentration: 132.73 ng/µl
Ratio[28S/18S]: 2.34

RQI: 9.1

Well# 8 Sample 8

Peak State	Peak Number	Mig. Time (secs)	Corrected Area	Comments
	1	21.30	1.42	
	2	25.16	3.48	
	3	26.55	1.56	
	4	39.08	13.76	
	5	41.68	1.04	
	6	42.31	2.03	
	7	44.29	37.17	

Egram, Gel Lane and Result Table Report

**Project:**	efra.muscle.rna	**Acq. Analyst:**	DefaultUser
**Assay:**	Eukaryote Total RNA StdSens	**Acq. Time:**	5/8/2021 8:32:00 PM
**Run:**	int-1.2.3rep_20-1.2rep_5-8-2021_8-31-59 PM	**Signature:**	N/A
**Run Version:**	N/A		

Well# 9 Sample 9

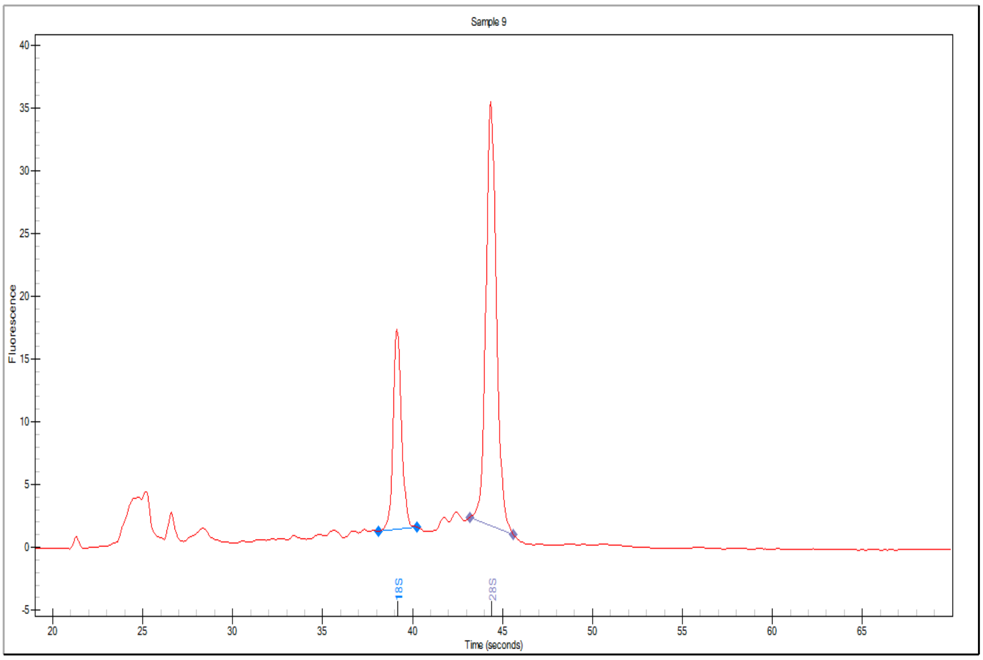

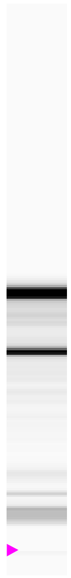

9

Well# 9 Sample 9

Fragment Number	Fragment Name	Start Time	End Time	Area	% of Total Area
1	18S	38.10	40.25	21.98	12.39
2	28S	43.20	45.60	51.48	29.01

RNA Area: 177.44
RNA Concentration: 211.53 ng/µl
Ratio[28S/18S]: 2.34

RQI: 9.4

Well# 9 Sample 9

Peak State	Peak Number	Mig. Time (secs)	Corrected Area	Comments
	1	21.30	1.29	
	2	25.18	8.38	
	3	26.59	3.64	
	4	28.34	2.30	
	5	39.16	22.64	
	6	41.73	1.42	
	7	42.45	2.65	

Project: efra.muscle.rna
Assay: Eukaryote Total RNA StdSens
Run: int-1.2.3rep_20-1.2rep_5-8-2021_8-31-59 PM
Run Version: N/A

Acq. Analyst: DefaultUser
Acq. Time: 5/8/2021 8:32:00 PM
Signature: N/A

Well# 9 Sample 9

Peak State	Peak Number	Mig. Time (secs)	Corrected Area	Comments
	8	44.35	58.37	

Project: efra.muscle.rna
Assay: Eukaryote Total RNA StdSens
Run: int-1.2.3rep_20-1.2rep_5-8-2021_8-31-59 PM
Run Version: N/A

Acq. Analyst: DefaultUser
Acq. Time: 5/8/2021 8:32:00 PM
Signature: N/A

Well# 10 Sample 10

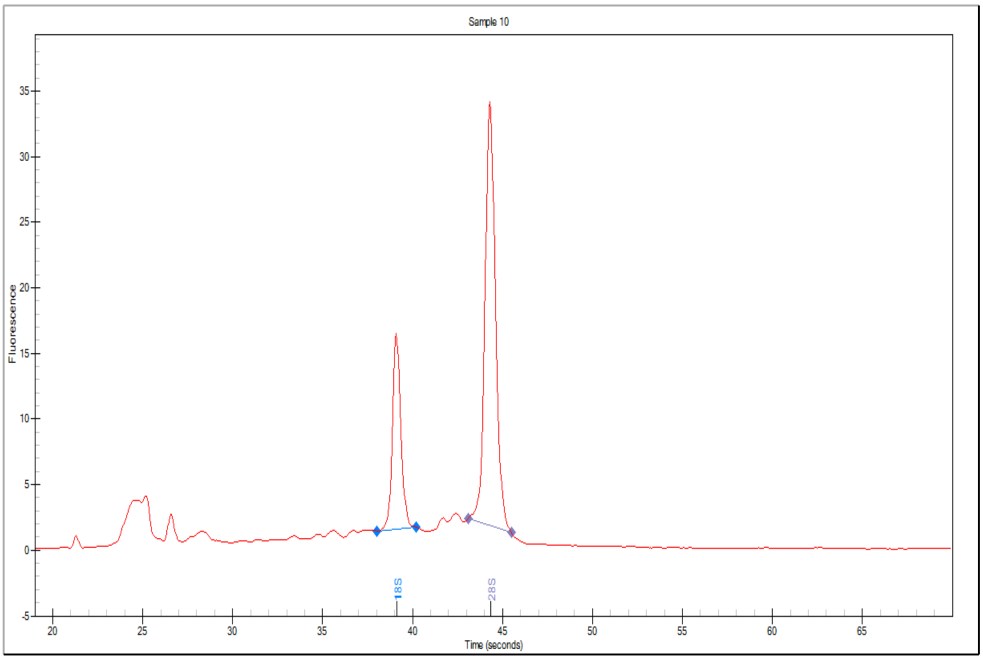

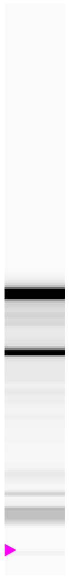

Well# 10 Sample 10

Fragment Number	Fragment Name	Start Time	End Time	Area	% of Total Area
1	18S	38.05	40.20	20.68	12.59
2	28S	43.15	45.55	49.11	29.89

RNA Area: 164.27
RNA Concentration: 195.83 ng/µl
Ratio[28S/18S]: 2.37

RQI: 9.2

Well# 10 Sample 10

Peak State	Peak Number	Mig. Time (secs)	Corrected Area	Comments
	1	21.30	1.30	
	2	24.54	12.72	
	3	25.17	7.08	
	4	26.58	3.27	
	5	39.11	20.53	
	6	41.68	1.20	
	7	42.41	2.44	

Egram, Gel Lane and Result Table Report

**Project:**	efra.muscle.rna	**Acq. Analyst:**	DefaultUser
**Assay:**	Eukaryote Total RNA StdSens	**Acq. Time:**	5/8/2021 8:32:00 PM
**Run:**	int-1.2.3rep_20-1.2rep_5-8-2021_8-31-59 PM	**Signature:**	N/A
**Run Version:**	N/A		

Well# 10 Sample 10

Peak State	Peak Number	Mig. Time (secs)	Corrected Area	Comments
	8	44.29	55.34	

Run Summary Report

**Project:**	efra.muscle.rna	**Acq. Analyst:**	DefaultUser
**Assay:**	Eukaryote Total RNA StdSens	**Acq. Time:**	5/8/2021 8:32:00 PM
**Run:**	int-1.2.3rep_20-1.2rep_5-8-2021_8-31-59 PM	**Signature:**	N/A
**Run Version:**	N/A		

Well ID	Sample Name	RNA Area	RNA Concentration (ng/µl)	Ratio [28S:18S]	RQI	RQI Classification	RQI Alert
L	Ladder	134.22	160.00				
1	Sample 1	990.54	1,180.83	2.11	9.9		
2	Sample 2	1,000.16	1,192.30	2.14	9.9		
3	Sample 3	440.10	524.65	2.12	8.2		
4	Sample 4	0.00	0.00	0.00	0.0		
5	Sample 5	496.17	591.49	2.17	8.4		
6	Sample 6	411.87	491.00	2.19	8.4		
7	Sample 7	128.65	153.36	2.32	9.0		
8	Sample 8	111.34	132.73	2.34	9.1		
9	Sample 9	177.44	211.53	2.34	9.4		
10	Sample 10	164.27	195.83	2.37	9.2		
