# Supplementary material for: Muscle Regeneration in Holothurians without the Upregulation of Muscle Genes
Source: Int J Mol Sci. 2022 Dec 16;23(24):16037. doi: 10.3390/ijms232416037 (PMC9785333; doi:10.3390/ijms232416037)
Supplement: Supplementary file 1 [file ijms-23-16037-s001.zip › Data/Data S2.pdf]

Run Overview Report

**Project:**	efra.muscle.rna	**Acq. Analyst:**	DefaultUser
**Assay:**	Eukaryote Total RNA StdSens	**Acq. Time:**	5/18/2021 9:20:33 PM
**Run:**	10d-1.2.3.4rep_5-18-2021_9-20-32 PM	**Signature:**	N/A
**Run Version:**	N/A		

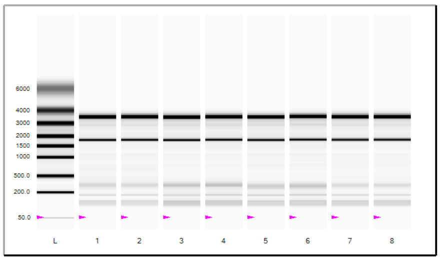

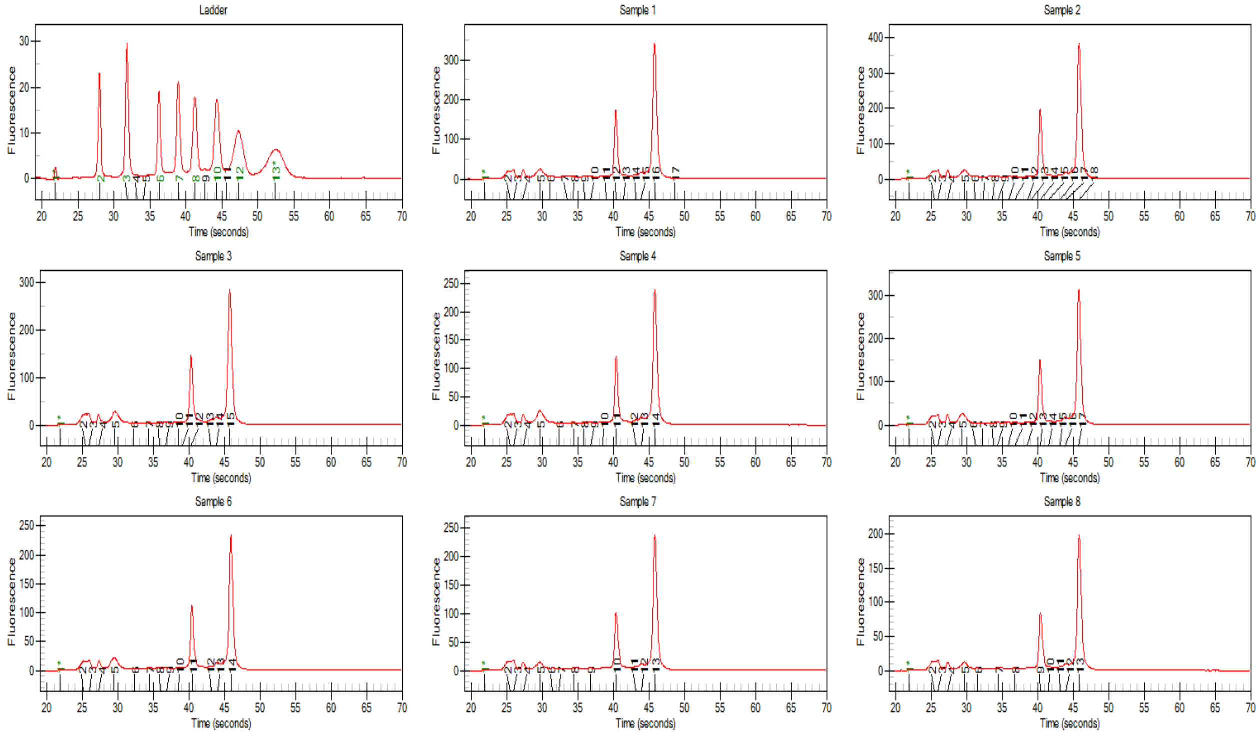

**Project:**	efra.muscle.rna	**Acq. Analyst:**	DefaultUser
**Assay:**	Eukaryote Total RNA StdSens	**Acq. Time:**	5/18/2021 9:20:33 PM
**Run:**	10d-1.2.3.4rep_5-18-2021_9-20-32 PM	**Signature:**	N/A
**Run Version:**	N/A		

Well# Ladder

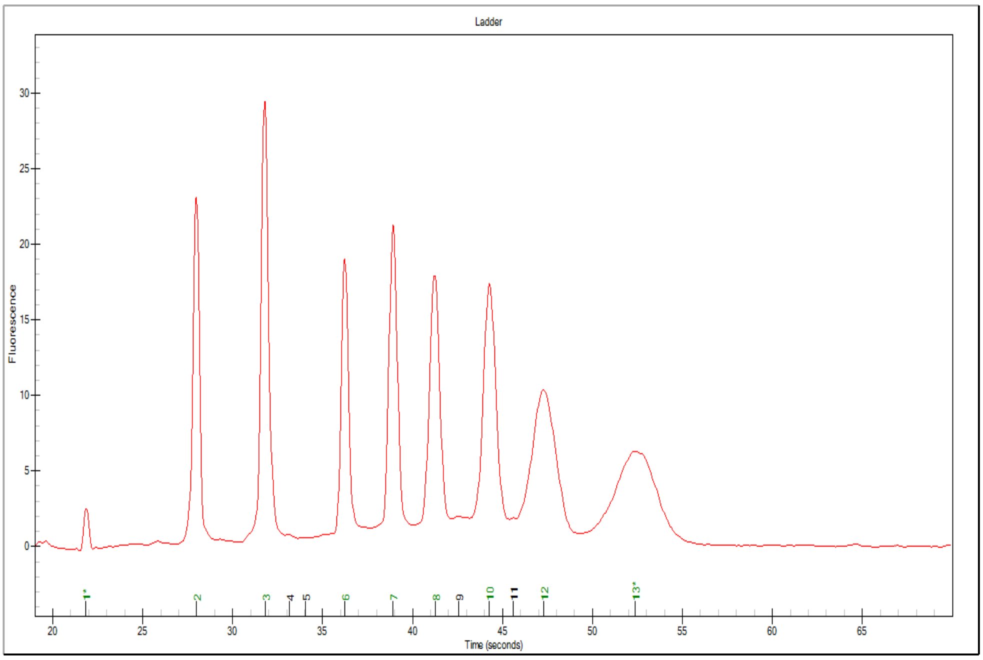

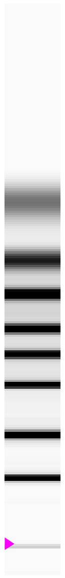

L

Well# Ladder

RNA Area: 303.41

RNA Concentration: 160.00 ng/µl

Well# Ladder

Peak State	Peak Number	Mig. Time (secs)	Corrected Area	Comments
	1	21.85	3.32	
L	2	28.00	37.68	
L	3	31.80	49.44	
	4	33.15	1.25	
	5	34.05	1.15	
L	6	36.25	29.09	
L	7	38.90	35.92	
L	8	41.25	33.94	
	9	42.55	2.89	
L	10	44.25	36.38	

Egram, Gel Lane and Result Table Report

**Project:**	efra.muscle.rna	**Acq. Analyst:**	DefaultUser
**Assay:**	Eukaryote Total RNA StdSens	**Acq. Time:**	5/18/2021 9:20:33 PM
**Run:**	10d-1.2.3.4rep_5-18-2021_9-20-32 PM	**Signature:**	N/A
**Run Version:**	N/A		

Well# Ladder

Peak State	Peak Number	Mig. Time (secs)	Corrected Area	Comments
	11	45.60	1.39	
L	12	47.30	37.03	
	13	52.35	34.12	

Egram, Gel Lane and Result Table Report

**Project:**	efra.muscle.rna	**Acq. Analyst:**	DefaultUser
**Assay:**	Eukaryote Total RNA StdSens	**Acq. Time:**	5/18/2021 9:20:33 PM
**Run:**	10d-1.2.3.4rep_5-18-2021_9-20-32 PM	**Signature:**	N/A
**Run Version:**	N/A		

Well# 3 Sample 3

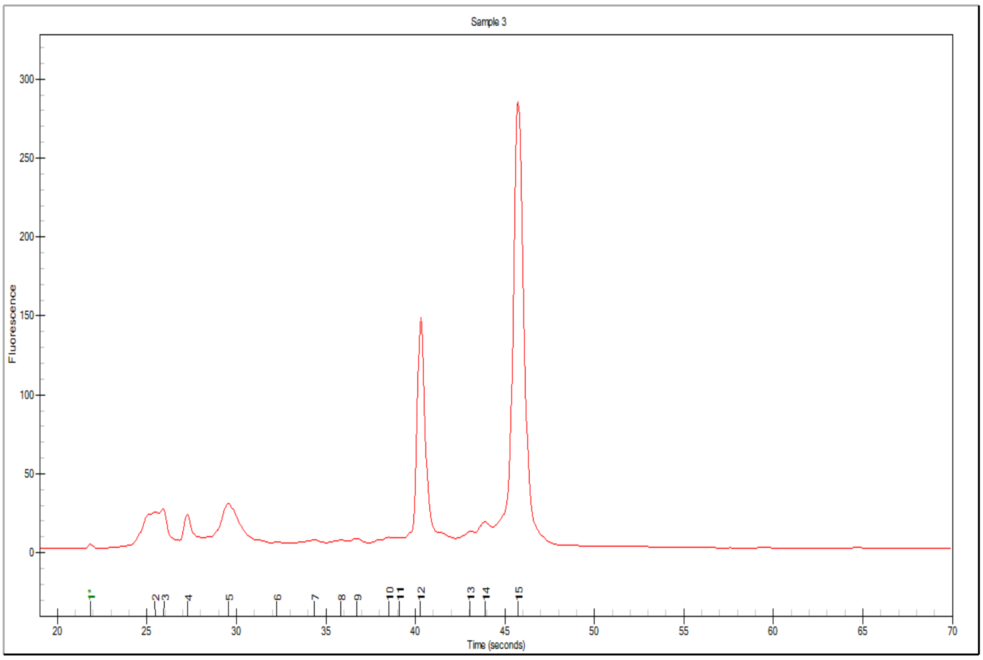

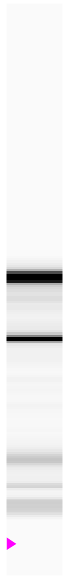

3

Well# 3 Sample 3					
Fragment Number	Fragment Name	Start Time	End Time	Area	% of Total Area
1	18S	39.30	41.40	174.00	13.77
2	28S	44.60	46.95	392.44	31.05

RNA Area:	1,264.05
RNA Concentration:	666.59 ng/µl
Ratio[28S/18S]:	2.26
RQI:	9.5

Well# 3 Sample 3				
**Peak State**	**Peak Number**	**Mig. Time (secs)**	**Corrected Area**	**Comments**
	1	21.85	3.60	
	2	25.47	87.30	
	3	25.91	52.16	
	4	27.28	45.39	
	5	29.57	112.12	
	6	32.26	1.04	
	7	34.36	3.98	

Project: efra.muscle.rna
Assay: Eukaryote Total RNA StdSens
Run: 10d-1.2.3.4rep_5-18-2021_9-20-32 PM
Run Version: N/A
Acq. Analyst: DefaultUser
Acq. Time: 5/18/2021 9:20:33 PM
Signature: N/A

Well# 3 Sample 3

Peak State	Peak Number	Mig. Time (secs)	Corrected Area	Comments
	8	35.83	4.34	
	9	36.71	6.01	
	10	38.52	5.72	
	11	39.11	4.38	
	12	40.28	214.37	
	13	43.06	15.02	
	14	43.90	26.36	
	15	45.75	461.18	

Project: efra.muscle.rna
Assay: Eukaryote Total RNA StdSens
Run: 10d-1.2.3.4rep_5-18-2021_9-20-32 PM
Run Version: N/A
Acq. Analyst: DefaultUser
Acq. Time: 5/18/2021 9:20:33 PM
Signature: N/A

Well# 4 Sample 4

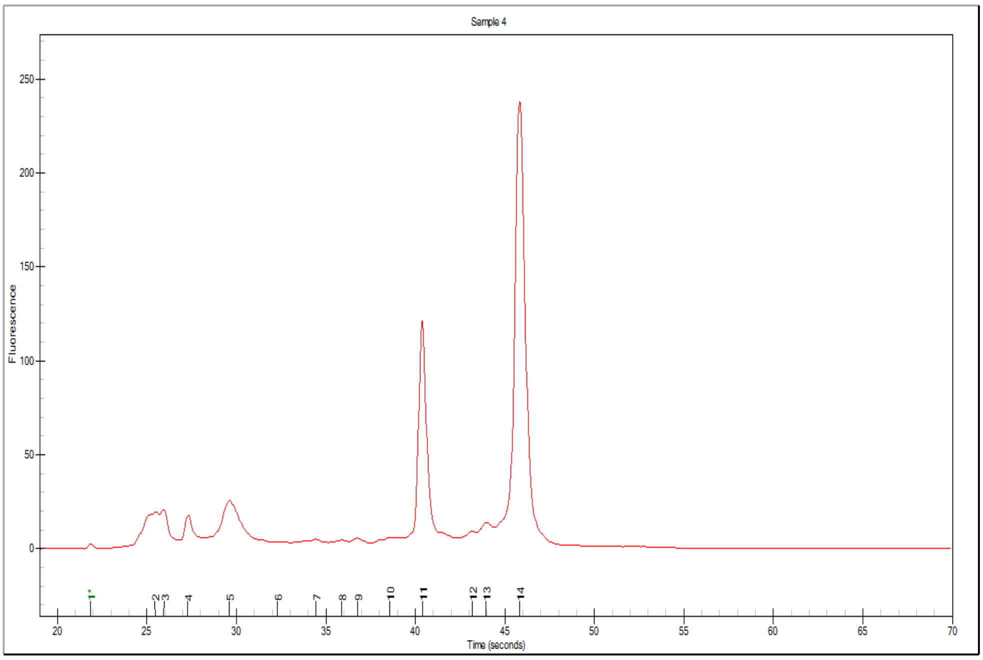

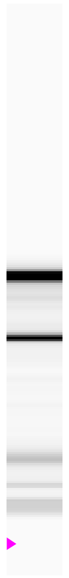

4

Well# 4 Sample 4

Fragment Number	Fragment Name	Start Time	End Time	Area	% of Total Area
1	18S	39.35	41.50	143.85	13.54
2	28S	44.70	47.05	326.69	30.74

RNA Area: 1,062.73
RNA Concentration: 560.43 ng/µl
Ratio[28S/18S]: 2.27

RQI: 9.4

Well# 4 Sample 4

Peak State	Peak Number	Mig. Time (secs)	Corrected Area	Comments
	1	21.85	3.42	
	2	25.49	74.37	
	3	25.93	41.85	
	4	27.29	37.97	
	5	29.62	101.09	
	6	32.29	0.78	
	7	34.43	3.11	

Project: efra.muscle.rna
Assay: Eukaryote Total RNA StdSens
Run: 10d-1.2.3.4rep_5-18-2021_9-20-32 PM
Run Version: N/A

Acq. Analyst: DefaultUser
Acq. Time: 5/18/2021 9:20:33 PM
Signature: N/A

Well# 4 Sample 4

Peak State	Peak Number	Mig. Time (secs)	Corrected Area	Comments
	8	35.88	2.80	
	9	36.76	4.50	
	10	38.55	8.56	
	11	40.40	175.61	
	12	43.17	12.09	
	13	43.94	21.39	
	14	45.84	382.01	

Project: efra.muscle.rna
Assay: Eukaryote Total RNA StdSens
Run: 10d-1.2.3.4rep_5-18-2021_9-20-32 PM
Run Version: N/A

Acq. Analyst: DefaultUser
Acq. Time: 5/18/2021 9:20:33 PM
Signature: N/A

Well# 5 Sample 5

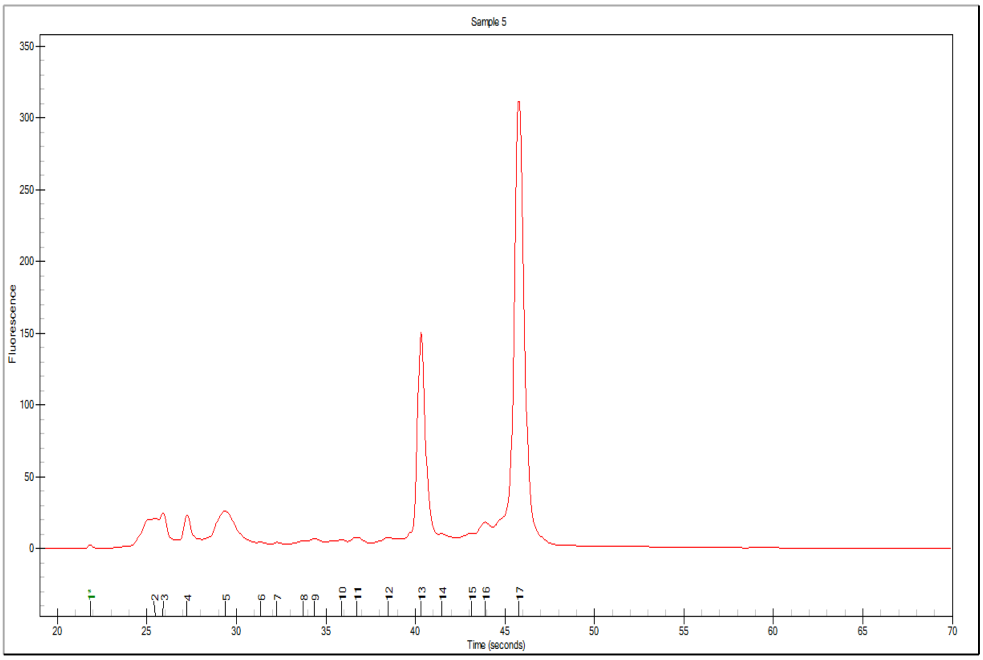

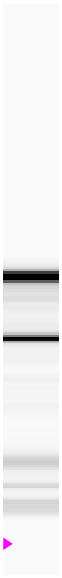

5

Well# 5 Sample 5					
Fragment Number	Fragment Name	Start Time	End Time	Area	% of Total Area
1	18S	39.30	41.30	181.97	14.18
2	28S	44.65	47.00	410.25	31.98

RNA Area: 1,282.83
RNA Concentration: 676.49 ng/µl
Ratio[28S/18S]: 2.25

RQI: 9.4

Well# 5 Sample 5				
Peak State	Peak Number	Mig. Time (secs)	Corrected Area	Comments
	1	21.85	2.89	
	2	25.46	80.57	
	3	25.89	55.80	
	4	27.24	50.90	
	5	29.36	127.40	
	6	31.33	5.93	
	7	32.25	7.77	

Egram, Gel Lane and Result Table Report

**Project:**	efra.muscle.rna	**Acq. Analyst:**	DefaultUser
**Assay:**	Eukaryote Total RNA StdSens	**Acq. Time:**	5/18/2021 9:20:33 PM
**Run:**	10d-1.2.3.4rep_5-18-2021_9-20-32 PM	**Signature:**	N/A
**Run Version:**	N/A		

Well# 5 Sample 5

Peak State	Peak Number	Mig. Time (secs)	Corrected Area	Comments
	8	33.74	8.95	
	9	34.36	13.36	
	10	35.86	7.38	
	11	36.72	15.41	
	12	38.45	21.85	
	13	40.33	214.89	
	14	41.49	15.57	
	15	43.12	17.20	
	16	43.89	29.67	
	17	45.77	487.10	

**Project:**	efra.muscle.rna	**Acq. Analyst:**	DefaultUser
**Assay:**	Eukaryote Total RNA StdSens	**Acq. Time:**	5/18/2021 9:20:33 PM
**Run:**	10d-1.2.3.4rep_5-18-2021_9-20-32 PM	**Signature:**	N/A
**Run Version:**	N/A		

Well# 6 Sample 6

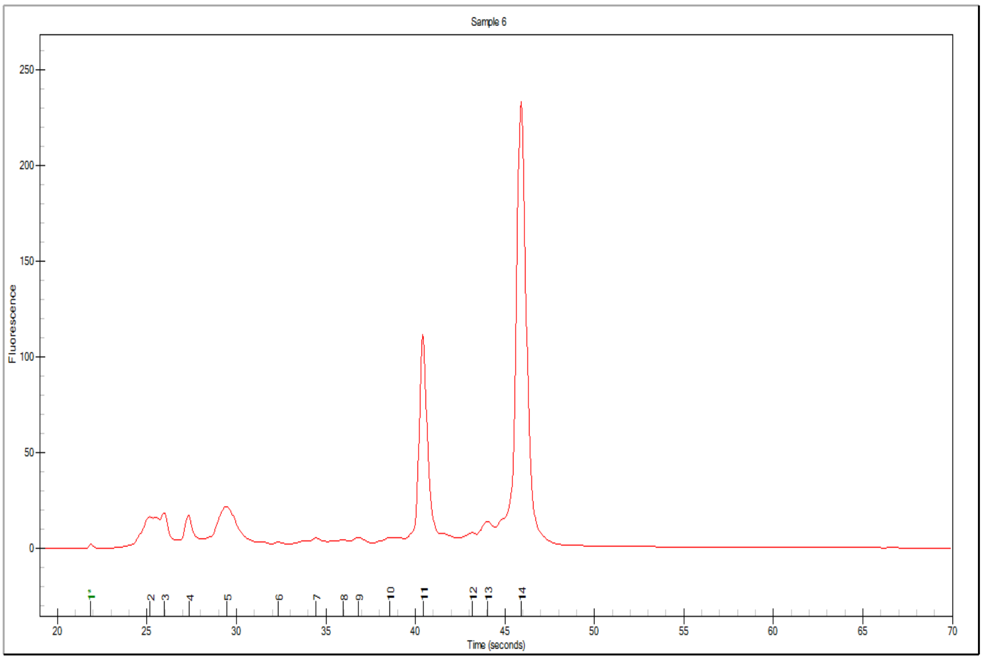

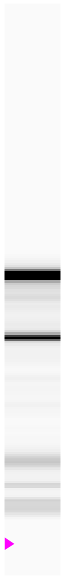

6

Well# 6 Sample 6

Fragment Number	Fragment Name	Start Time	End Time	Area	% of Total Area
1	18S	39.40	41.40	135.03	13.76
2	28S	44.80	47.10	303.80	30.96

RNA Area: 981.20
RNA Concentration: 517.43 ng/µl
Ratio[28S/18S]: 2.25

RQI: 9.4

Well# 6 Sample 6

Peak State	Peak Number	Mig. Time (secs)	Corrected Area	Comments
	1	21.85	2.70	
	2	25.17	48.95	
	3	25.99	39.50	
	4	27.34	39.68	
	5	29.45	110.86	
	6	32.34	6.35	
	7	34.46	10.34	

Egram, Gel Lane and Result Table Report

**Project:**	efra.muscle.rna	**Acq. Analyst:**	DefaultUser
**Assay:**	Eukaryote Total RNA StdSens	**Acq. Time:**	5/18/2021 9:20:33 PM
**Run:**	10d-1.2.3.4rep_5-18-2021_9-20-32 PM	**Signature:**	N/A
**Run Version:**	N/A		

Well# 6 Sample 6

Peak State	Peak Number	Mig. Time (secs)	Corrected Area	Comments
	8	35.95	5.19	
	9	36.82	11.42	
	10	38.55	16.45	
	11	40.43	162.84	
	12	43.17	11.94	
	13	44.04	22.44	
	14	45.91	363.10	

Project: efra.muscle.rna
Assay: Eukaryote Total RNA StdSens
Run: 10d-1.2.3.4rep_5-18-2021_9-20-32 PM
Run Version: N/A

Acq. Analyst: DefaultUser
Acq. Time: 5/18/2021 9:20:33 PM
Signature: N/A

Well# 7 Sample 7

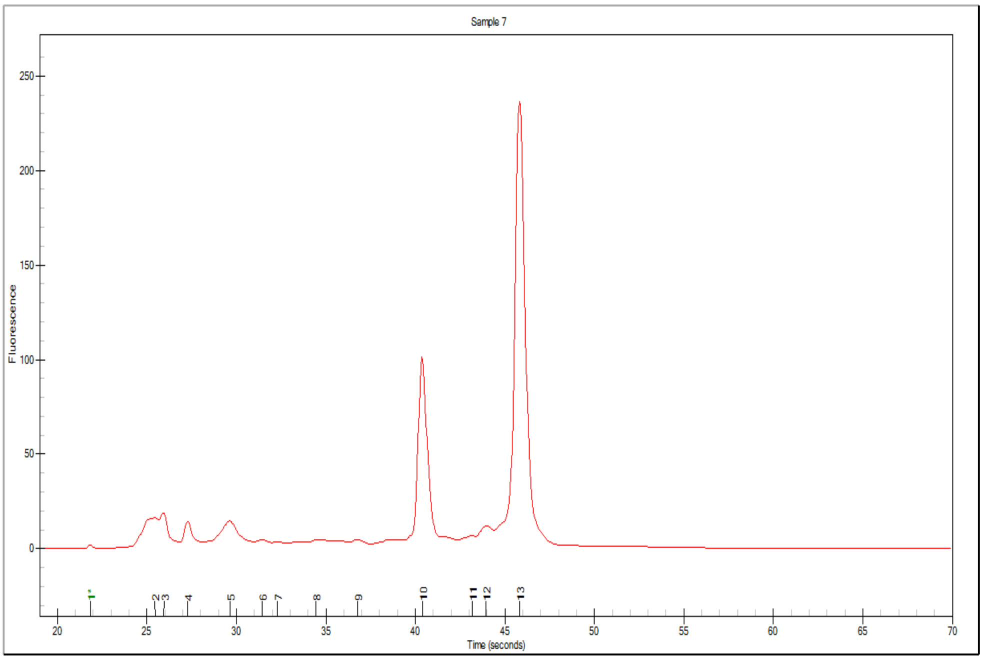

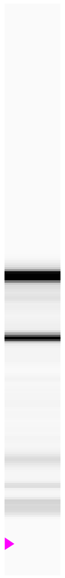

7

**Well# 7 Sample 7**					
Fragment Number	Fragment Name	Start Time	End Time	Area	% of Total Area
1	18S	39.30	41.35	137.39	14.68
2	28S	44.70	47.05	321.59	34.37

RNA Area: 935.72
RNA Concentration: 493.45 ng/µl
Ratio[28S/18S]: 2.34

RQI: 9.3

**Well# 7 Sample 7**				
Peak State	Peak Number	Mig. Time (secs)	Corrected Area	Comments
	1	21.85	2.64	
	2	25.46	58.09	
	3	25.94	39.05	
	4	27.29	26.22	
	5	29.65	45.96	
	6	31.43	3.35	
	7	32.29	0.61	

**Project:**	efra.muscle.rna	**Acq. Analyst:**	DefaultUser
**Assay:**	Eukaryote Total RNA StdSens	**Acq. Time:**	5/18/2021 9:20:33 PM
**Run:**	10d-1.2.3.4rep_5-18-2021_9-20-32 PM	**Signature:**	N/A
**Run Version:**	N/A		

Well# 7 Sample 7

Peak State	Peak Number	Mig. Time (secs)	Corrected Area	Comments
	8	34.46	1.01	
	9	36.77	0.51	
	10	40.38	149.09	
	11	43.17	5.71	
	12	43.94	15.86	
	13	45.82	371.42	

Egram, Gel Lane and Result Table Report

Project: efra.muscle.rna
Assay: Eukaryote Total RNA StdSens
Run: 10d-1.2.3.4rep_5-18-2021_9-20-32 PM
Run Version: N/A

Acq. Analyst: DefaultUser
Acq. Time: 5/18/2021 9:20:33 PM
Signature: N/A

Well# 8 Sample 8

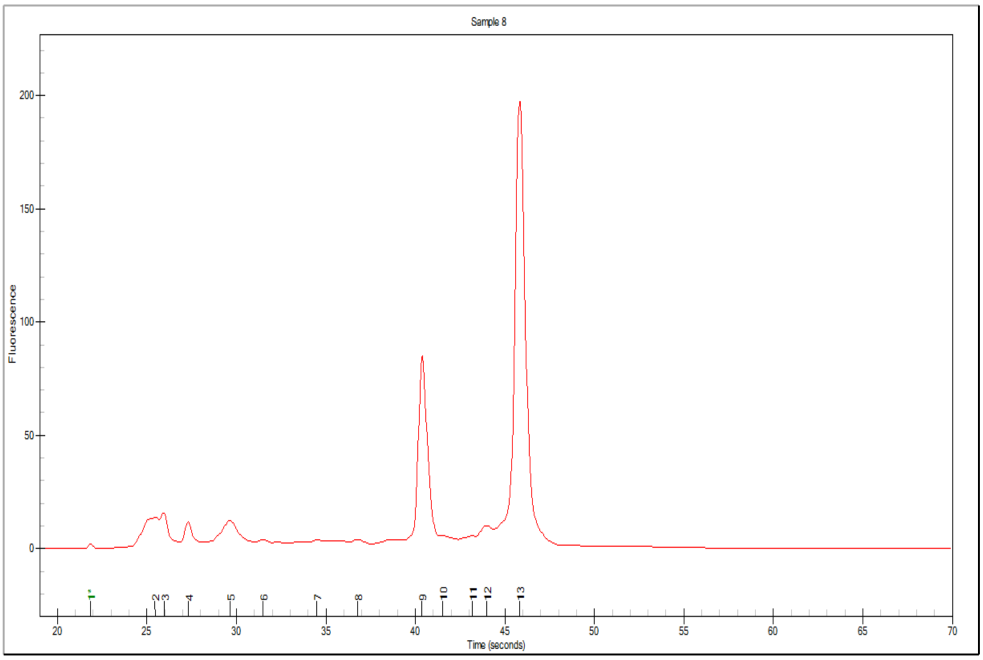

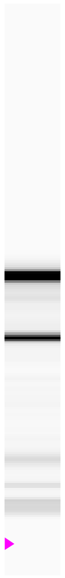

8

Well# 8 Sample 8					
Fragment Number	Fragment Name	Start Time	End Time	Area	% of Total Area
1	18S	39.35	41.35	114.56	14.54
2	28S	44.70	47.05	269.35	34.18

RNA Area: 787.93
RNA Concentration: 415.51 ng/µl
Ratio[28S/18S]: 2.35

RQI: 9.3

Well# 8 Sample 8				
Peak State	Peak Number	Mig. Time (secs)	Corrected Area	Comments
	1	21.85	2.57	
	2	25.48	47.64	
	3	25.96	31.59	
	4	27.31	20.40	
	5	29.63	39.33	
	6	31.47	2.56	
	7	34.47	0.82	

Egram, Gel Lane and Result Table Report

Project: efra.muscle.rna
Assay: Eukaryote Total RNA StdSens
Run: 10d-1.2.3.4rep_5-18-2021_9-20-32 PM
Run Version: N/A

Acq. Analyst: DefaultUser
Acq. Time: 5/18/2021 9:20:33 PM
Signature: N/A

Well# 8 Sample 8

Peak State	Peak Number	Mig. Time (secs)	Corrected Area	Comments
	8	36.79	0.43	
	9	40.36	120.24	
	10	41.52	4.55	
	11	43.17	4.65	
	12	43.99	13.05	
	13	45.83	312.28	

Run Summary Report

Project: efra.muscle.rna
Assay: Eukaryote Total RNA StdSens
Run: 10d-1.2.3.4rep_5-18-2021_9-20-32 PM
Run Version: N/A

Acq. Analyst: DefaultUser
Acq. Time: 5/18/2021 9:20:33 PM
Signature: N/A

Well ID	Sample Name	RNA Area	RNA Concentration (ng/µl)	Ratio [28S:18S]	RQI	RQI Classification	RQI Alert
L	Ladder	303.41	160.00				
1	Sample 1	1,408.66	742.85	2.26	9.1	■	
2	Sample 2	1,539.56	811.88	2.26	9.2	■	
3	Sample 3	1,264.05	666.59	2.26	9.5	■	
4	Sample 4	1,062.73	560.43	2.27	9.4	■	
5	Sample 5	1,282.83	676.49	2.25	9.4	■	
6	Sample 6	981.20	517.43	2.25	9.4	■	
7	Sample 7	935.72	493.45	2.34	9.3	■	
8	Sample 8	787.93	415.51	2.35	9.3	■	
