# Supplementary material for: Muscle Regeneration in Holothurians without the Upregulation of Muscle Genes
Source: Int J Mol Sci. 2022 Dec 16;23(24):16037. doi: 10.3390/ijms232416037 (PMC9785333; doi:10.3390/ijms232416037)
Supplement: Supplementary file 1 [file ijms-23-16037-s001.zip › Data/Data S3.pdf]

Run Overview Report

**Project:**	efra.muscle.rna	**Acq. Analyst:**	DefaultUser
**Assay:**	Eukaryote Total RNA StdSens	**Acq. Time:**	3/17/2021 12:42:55 PM
**Run:**	Run_3-17-2021_12-42-53 PM	**Signature:**	N/A
**Run Version:**	N/A		

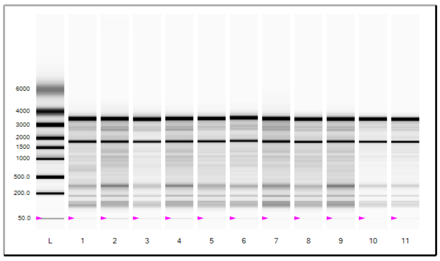

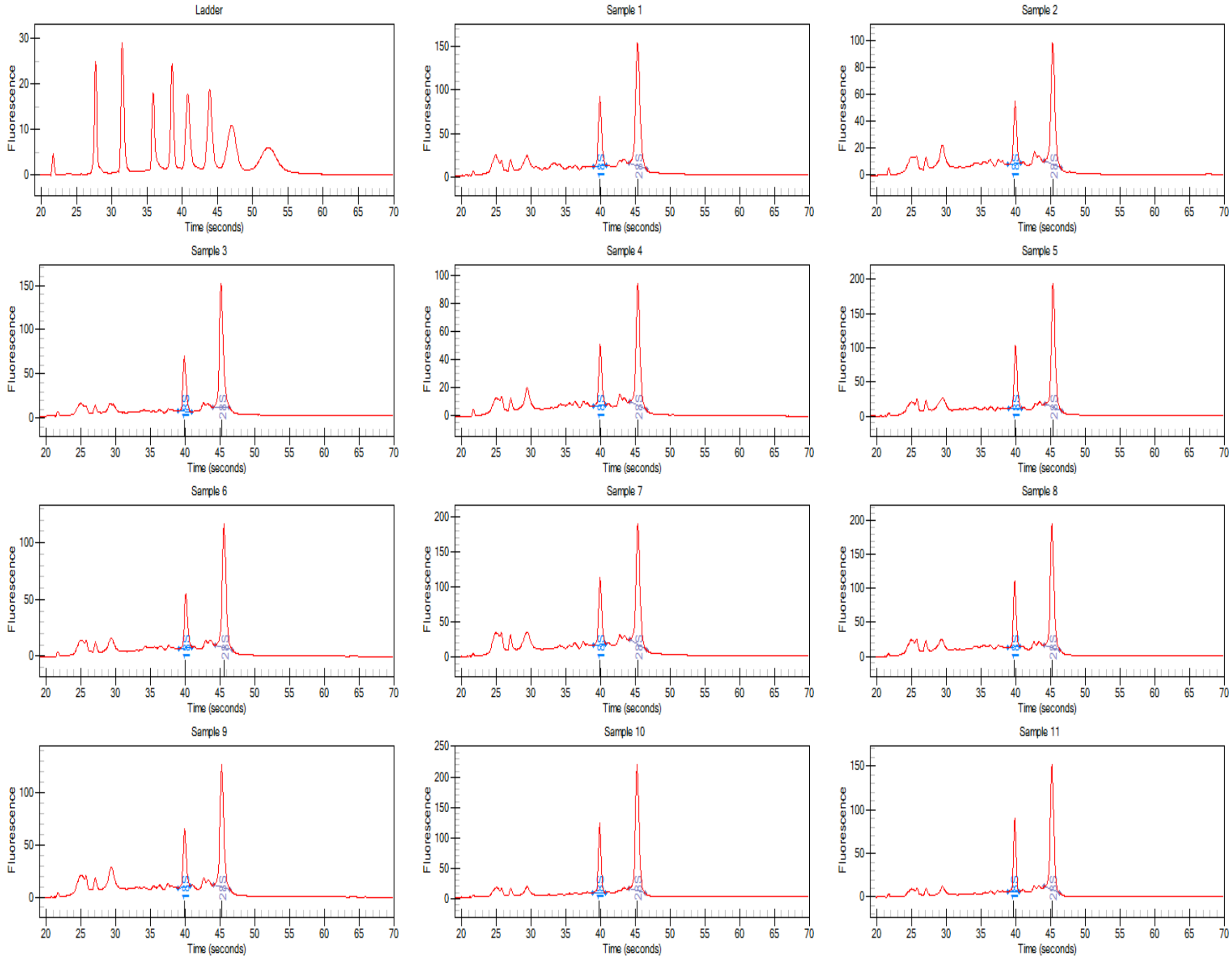

Project: efra.muscle.rna
Assay: Eukaryote Total RNA StdSens
Run: Run_3-17-2021_12-42-53 PM
Run Version: N/A

Acq. Analyst: DefaultUser
Acq. Time: 3/17/2021 12:42:55 PM
Signature: N/A

Well# Ladder

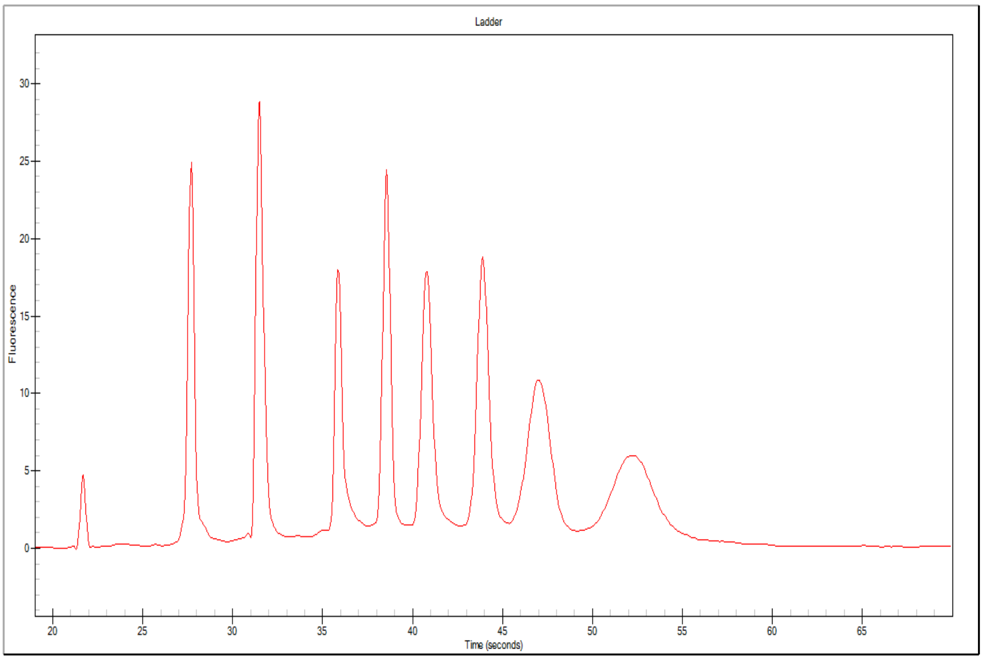

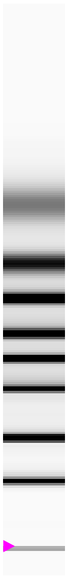

L

Well# Ladder

RNA Area: 326.67
RNA Concentration: 160.00 ng/µl

Well# Ladder

Peak State	Peak Number	Mig. Time (secs)	Corrected Area	Comments
	1	21.70	7.33	
L	2	27.70	44.00	
	3	30.85	2.06	
L	4	31.50	48.15	
	5	33.65	1.83	
	6	35.20	2.32	
L	7	35.85	30.43	
L	8	38.55	37.41	
L	9	40.80	36.85	
L	10	43.90	37.19	

Project: efra.muscle.rna
Assay: Eukaryote Total RNA StdSens
Run: Run_3-17-2021_12-42-53 PM
Run Version: N/A

Acq. Analyst: DefaultUser
Acq. Time: 3/17/2021 12:42:55 PM
Signature: N/A

Well# Ladder

Peak State	Peak Number	Mig. Time (secs)	Corrected Area	Comments
L	11	47.00	37.74	
	12	52.25	31.61	

Egram, Gel Lane and Result Table Report

Project: efra.muscle.rna
Assay: Eukaryote Total RNA StdSens
Run: Run_3-17-2021_12-42-53 PM
Run Version: N/A

Acq. Analyst: DefaultUser
Acq. Time: 3/17/2021 12:42:55 PM
Signature: N/A

Well# 2 Sample 2

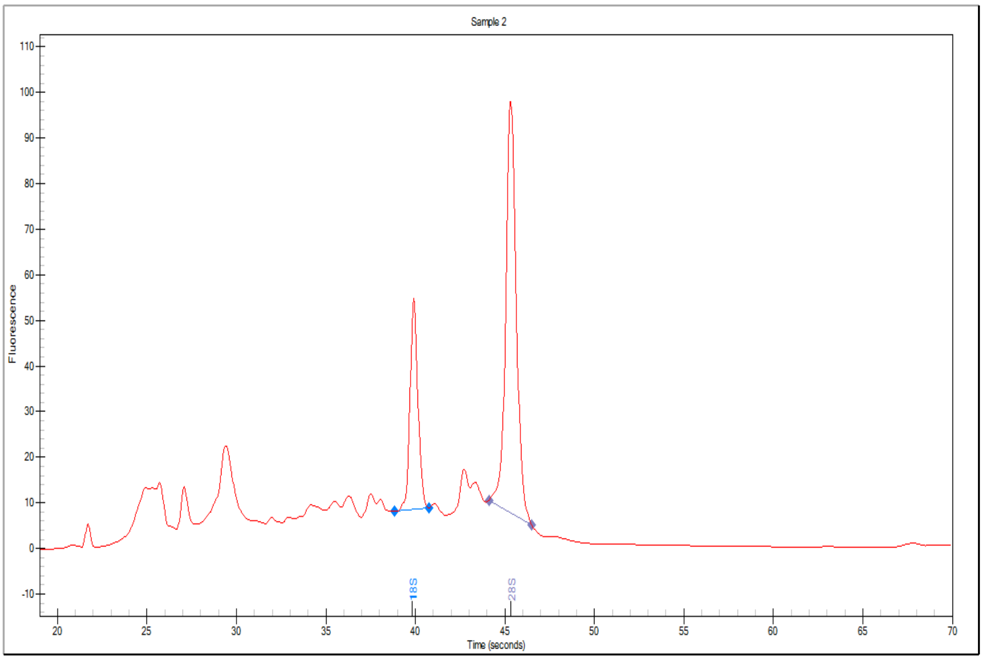

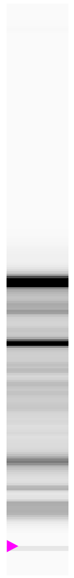

Well# 2 Sample 2

Fragment Number	Fragment Name	Start Time	End Time	Area	% of Total Area
1	18S	38.90	40.75	59.50	7.27
2	28S	44.15	46.55	135.47	16.56

RNA Area: 818.04
RNA Concentration: 400.66 ng/µl
Ratio[28S/18S]: 2.28

RQI: 8.1

Well# 2 Sample 2

Peak State	Peak Number	Mig. Time (secs)	Corrected Area	Comments
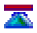	1	21.70	5.11	
	2	24.93	39.17	
	3	25.72	32.30	
	4	27.09	25.85	
	5	29.39	106.48	
	6	31.99	12.56	
	7	32.97	8.99	

Project: efra.muscle.rna
Assay: Eukaryote Total RNA StdSens
Run: Run_3-17-2021_12-42-53 PM
Run Version: N/A

Acq. Analyst: DefaultUser
Acq. Time: 3/17/2021 12:42:55 PM
Signature: N/A

Well# 2 Sample 2

Peak State	Peak Number	Mig. Time (secs)	Corrected Area	Comments
	8	34.19	22.25	
	9	35.51	17.85	
	10	36.30	23.43	
	11	37.52	16.53	
	12	38.06	18.82	
	13	39.92	87.81	
	14	41.05	13.93	
	15	42.71	27.74	
	16	43.35	19.96	
	17	45.31	168.43	
	18	67.79	0.96	

Project: efra.muscle.rna
Assay: Eukaryote Total RNA StdSens
Run: Run_3-17-2021_12-42-53 PM
Run Version: N/A

Acq. Analyst: DefaultUser
Acq. Time: 3/17/2021 12:42:55 PM
Signature: N/A

Well# 3 Sample 3

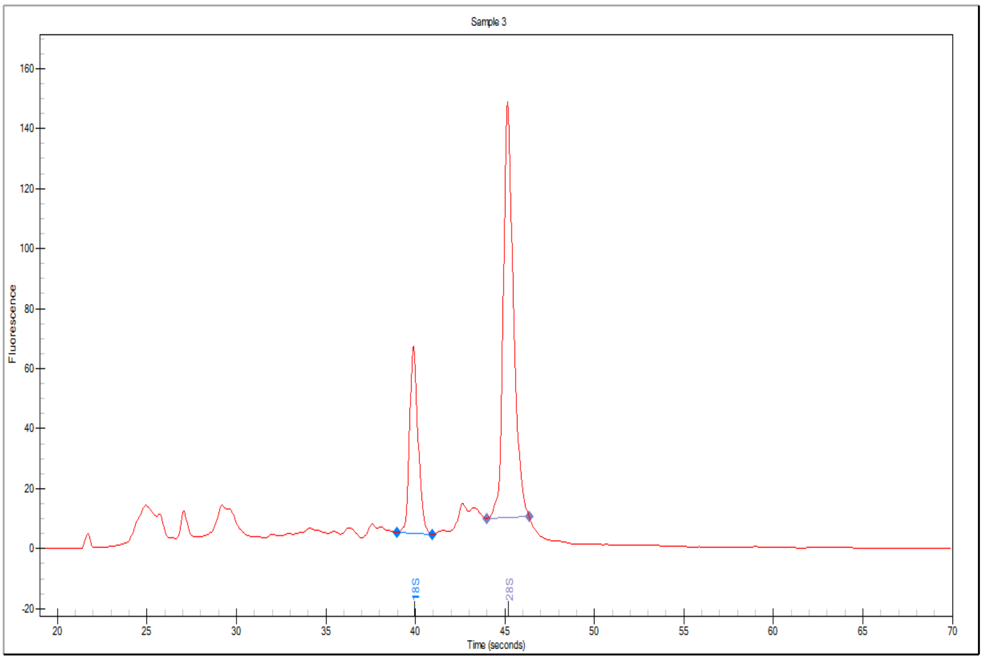

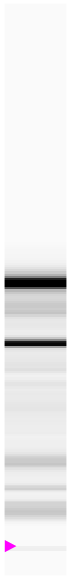

3

Well# 3 Sample 3					
Fragment Number	Fragment Name	Start Time	End Time	Area	% of Total Area
1	18S	38.95	40.95	86.15	10.65
2	28S	44.00	46.40	203.86	25.21

RNA Area: 808.72
RNA Concentration: 396.10 ng/µl
Ratio[28S/18S]: 2.37

RQI: 8.5

Well# 3 Sample 3				
Peak State	Peak Number	Mig. Time (secs)	Corrected Area	Comments
	1	21.70	6.98	
	2	24.95	62.31	
	3	25.72	24.55	
	4	27.08	26.84	
	5	29.21	75.96	
	6	32.02	9.44	
	7	32.94	5.66	

Project: efra.muscle.rna
Assay: Eukaryote Total RNA StdSens
Run: Run_3-17-2021_12-42-53 PM
Run Version: N/A

Acq. Analyst: DefaultUser
Acq. Time: 3/17/2021 12:42:55 PM
Signature: N/A

Well# 3 Sample 3

Peak State	Peak Number	Mig. Time (secs)	Corrected Area	Comments
	8	34.10	18.06	
	9	35.46	7.26	
	10	36.33	12.55	
	11	37.59	9.97	
	12	38.07	12.38	
	13	39.86	95.53	
	14	41.56	7.53	
	15	42.67	21.20	
	16	43.30	22.31	
	17	45.14	250.56	

Project: efra.muscle.rna
Assay: Eukaryote Total RNA StdSens
Run: Run_3-17-2021_12-42-53 PM
Run Version: N/A

Acq. Analyst: DefaultUser
Acq. Time: 3/17/2021 12:42:55 PM
Signature: N/A

Well# 5 Sample 5

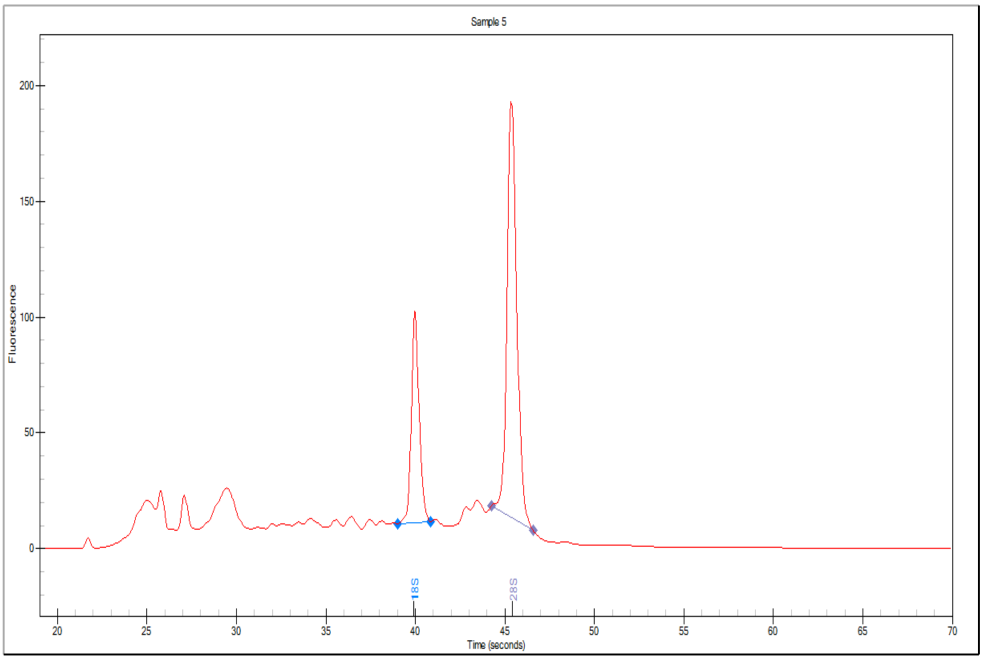

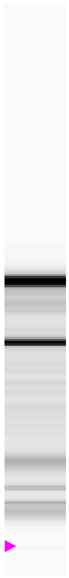

5

**Well# 5 Sample 5**					
Fragment Number	Fragment Name	Start Time	End Time	Area	% of Total Area
1	18S	39.00	40.85	115.88	9.15
2	28S	44.25	46.60	250.55	19.79

RNA Area: 1,265.94
RNA Concentration: 620.04 ng/µl
Ratio[28S/18S]: 2.16

RQI: 8.2

**Well# 5 Sample 5**				
Peak State	Peak Number	Mig. Time (secs)	Corrected Area	Comments
	1	21.70	3.80	
	2	25.02	95.37	
	3	25.77	57.91	
	4	27.10	52.33	
	5	29.47	139.15	
	6	31.22	17.27	
	7	32.03	15.57	

Project: efra.muscle.rna
Assay: Eukaryote Total RNA StdSens
Run: Run_3-17-2021_12-42-53 PM
Run Version: N/A

Acq. Analyst: DefaultUser
Acq. Time: 3/17/2021 12:42:55 PM
Signature: N/A

Well# 5 Sample 5

Peak State	Peak Number	Mig. Time (secs)	Corrected Area	Comments
	8	32.55	20.94	
	9	33.45	16.51	
	10	34.11	31.21	
	11	35.58	23.24	
	12	36.44	26.74	
	13	37.43	18.90	
	14	38.14	14.60	
	15	38.76	10.59	
	16	39.99	156.31	
	17	41.13	18.39	
	18	42.83	30.43	
	19	43.45	31.66	
	20	45.34	317.01	
	21	48.37	0.62	

Egram, Gel Lane and Result Table Report

**Project:**	efra.muscle.rna	**Acq. Analyst:**	DefaultUser
**Assay:**	Eukaryote Total RNA StdSens	**Acq. Time:**	3/17/2021 12:42:55 PM
**Run:**	Run_3-17-2021_12-42-53 PM	**Signature:**	N/A
**Run Version:**	N/A		

Well# 7 Sample 7

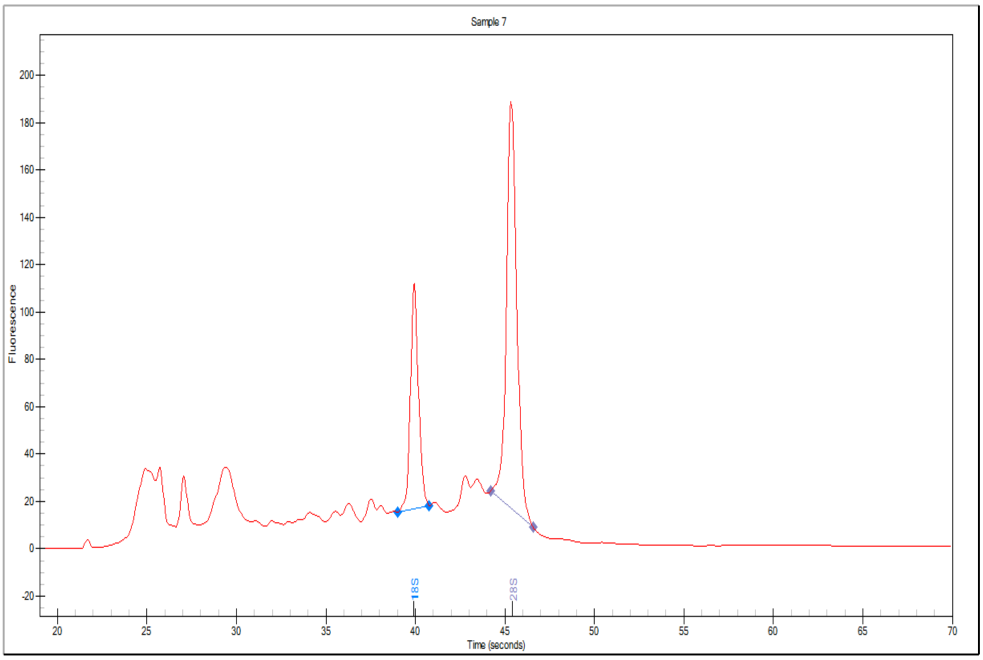

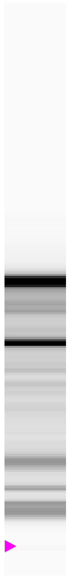

7

Well# 7 Sample 7

Fragment Number	Fragment Name	Start Time	End Time	Area	% of Total Area
1	18S	39.00	40.80	125.39	7.88
2	28S	44.20	46.60	254.91	16.02

RNA Area: 1,591.45
RNA Concentration: 779.46 ng/µl
Ratio[28S/18S]: 2.03

RQI: 8.3

Well# 7 Sample 7

Peak State	Peak Number	Mig. Time (secs)	Corrected Area	Comments
	1	21.70	1.59	
	2	24.96	137.45	
	3	25.71	74.77	
	4	27.08	67.98	
	5	29.39	168.84	
	6	31.04	19.76	
	7	31.98	24.54	

Egram, Gel Lane and Result Table Report

Project: efra.muscle.rna
Assay: Eukaryote Total RNA StdSens
Run: Run_3-17-2021_12-42-53 PM
Run Version: N/A
Acq. Analyst: DefaultUser
Acq. Time: 3/17/2021 12:42:55 PM
Signature: N/A

Well# 7 Sample 7

Peak State	Peak Number	Mig. Time (secs)	Corrected Area	Comments
	8	32.93	12.75	
	9	34.11	36.93	
	10	35.52	26.14	
	11	36.28	38.60	
	12	37.50	30.92	
	13	38.07	20.00	
	14	38.78	16.26	
	15	39.91	185.36	
	16	41.09	30.85	
	17	42.79	56.53	
	18	43.45	44.82	
	19	45.33	334.70	

Project: efra.muscle.rna
Assay: Eukaryote Total RNA StdSens
Run: Run_3-17-2021_12-42-53 PM
Run Version: N/A

Acq. Analyst: DefaultUser
Acq. Time: 3/17/2021 12:42:55 PM
Signature: N/A

Well# 10 Sample 10

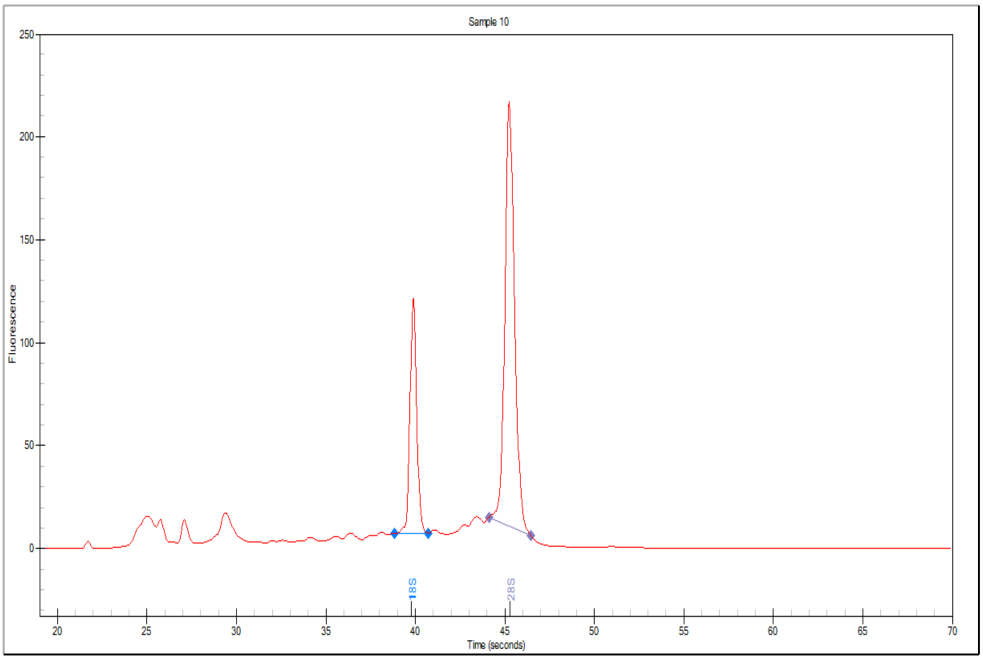

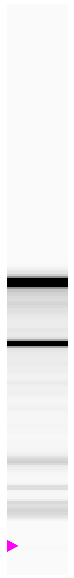

Well# 10 Sample 10					
Fragment Number	Fragment Name	Start Time	End Time	Area	% of Total Area
1	18S	38.85	40.70	126.08	13.89
2	28S	44.15	46.45	283.84	31.26

RNA Area: 907.99
RNA Concentration: 444.72 ng/µl
Ratio[28S/18S]: 2.25

RQI: 9.3

Well# 10 Sample 10				
Peak State	Peak Number	Mig. Time (secs)	Corrected Area	Comments
	1	21.70	4.79	
	2	25.00	65.66	
	3	25.76	29.48	
	4	27.10	26.56	
	5	29.40	73.49	
	6	32.02	5.06	
	7	32.55	7.52	

Egram, Gel Lane and Result Table Report

**Project:**	efra.muscle.rna	**Acq. Analyst:**	DefaultUser
**Assay:**	Eukaryote Total RNA StdSens	**Acq. Time:**	3/17/2021 12:42:55 PM
**Run:**	Run_3-17-2021_12-42-53 PM	**Signature:**	N/A
**Run Version:**	N/A		

Well# 10 Sample 10

Peak State	Peak Number	Mig. Time (secs)	Corrected Area	Comments
	8	34.13	12.04	
	9	35.56	12.31	
	10	36.37	15.24	
	11	37.52	9.53	
	12	38.09	12.00	
	13	39.86	153.75	
	14	41.06	17.24	
	15	42.73	20.26	
	16	43.40	25.33	
	17	45.26	346.83	

Project: efra.muscle.rna
Assay: Eukaryote Total RNA StdSens
Run: Run_3-17-2021_12-42-53 PM
Run Version: N/A

Acq. Analyst: DefaultUser
Acq. Time: 3/17/2021 12:42:55 PM
Signature: N/A

Well# 11 Sample 11

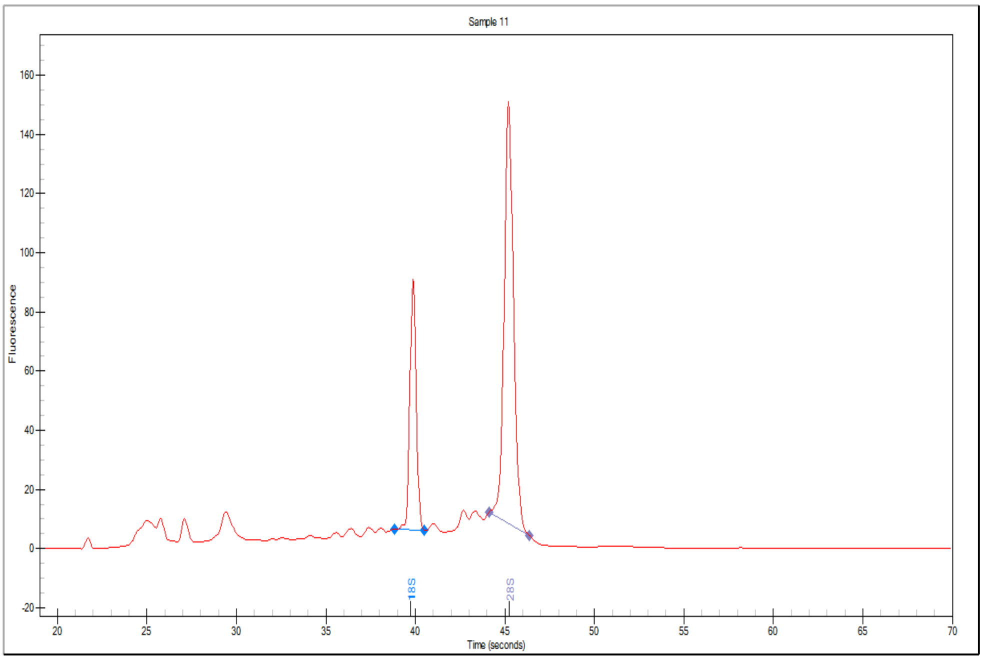

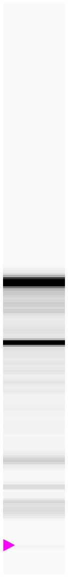

Well# 11 Sample 11

Fragment Number	Fragment Name	Start Time	End Time	Area	% of Total Area
1	18S	38.90	40.55	88.55	13.20
2	28S	44.10	46.40	191.59	28.55

RNA Area: 671.02
RNA Concentration: 328.66 ng/µl
Ratio[28S/18S]: 2.16

RQI: 9.1

Well# 11 Sample 11

Peak State	Peak Number	Mig. Time (secs)	Corrected Area	Comments
	1	21.70	4.81	
	2	25.02	36.00	
	3	25.79	22.15	
	4	27.09	19.44	
	5	29.40	41.39	
	6	31.08	5.48	
	7	32.00	3.95	

Egram, Gel Lane and Result Table Report

Project: efra.muscle.rna
Assay: Eukaryote Total RNA StdSens
Run: Run_3-17-2021_12-42-53 PM
Run Version: N/A

Acq. Analyst: DefaultUser
Acq. Time: 3/17/2021 12:42:55 PM
Signature: N/A

Well# 11 Sample 11

Peak State	Peak Number	Mig. Time (secs)	Corrected Area	Comments
	8	32.53	7.00	
	9	34.11	10.49	
	10	35.56	8.65	
	11	36.38	12.89	
	12	37.39	11.16	
	13	38.06	8.90	
	14	39.31	6.50	
	15	39.89	103.22	
	16	40.99	14.89	
	17	42.68	23.78	
	18	43.35	18.44	
	19	45.18	237.29	

Run Summary Report

Project: efra.muscle.rna
Assay: Eukaryote Total RNA StdSens
Run: Run_3-17-2021_12-42-53 PM
Run Version: N/A

Acq. Analyst: DefaultUser
Acq. Time: 3/17/2021 12:42:55 PM
Signature: N/A

Well ID	Sample Name	RNA Area	RNA Concentration (ng/µl)	Ratio [28S:18S]	RQI	RQI Classification	RQI Alert
L	Ladder	326.67	160.00				
1	Sample 1	1,111.14	544.22	2.15	8.2		
2	Sample 2	818.04	400.66	2.28	8.1		
3	Sample 3	808.72	396.10	2.37	8.5		
4	Sample 4	751.73	368.18	2.27	8.1		
5	Sample 5	1,265.94	620.04	2.16	8.2		
6	Sample 6	779.73	381.90	2.45	8.2		
7	Sample 7	1,591.45	779.46	2.03	8.3		
8	Sample 8	1,324.53	648.73	2.23	8.1		
9	Sample 9	1,017.98	498.59	2.34	8.1		
10	Sample 10	907.99	444.72	2.25	9.3		
11	Sample 11	671.02	328.66	2.16	9.1		
