# Supplementary figures and images for: Muscle Regeneration in Holothurians without the Upregulation of Muscle Genes
Source: Int J Mol Sci. 2022 Dec 16;23(24):16037. doi: 10.3390/ijms232416037 (PMC9785333; doi:10.3390/ijms232416037)

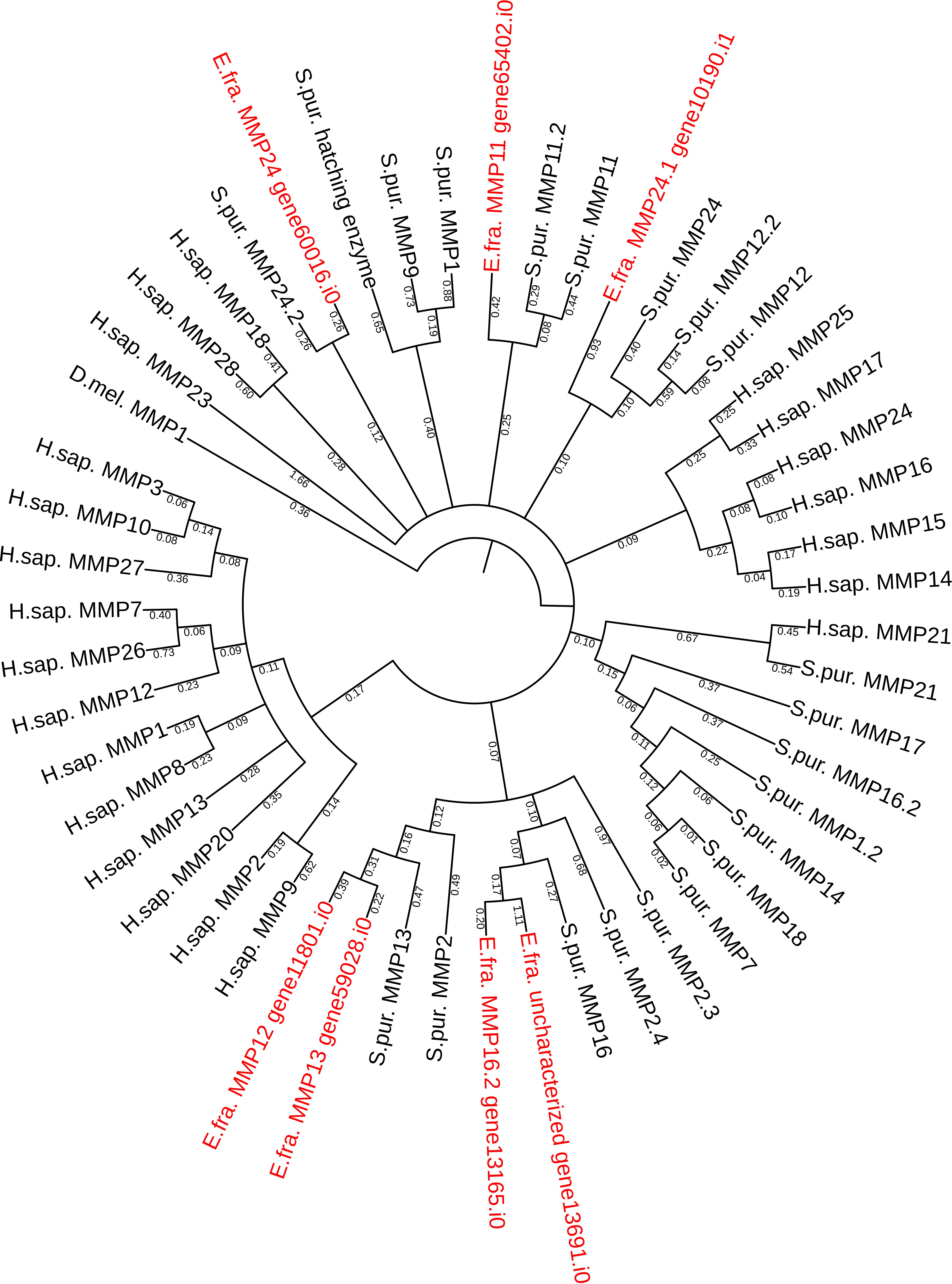

Supplement: Supplementary file 1 [file ijms-23-16037-s001.zip › Data/Figure S1.png]

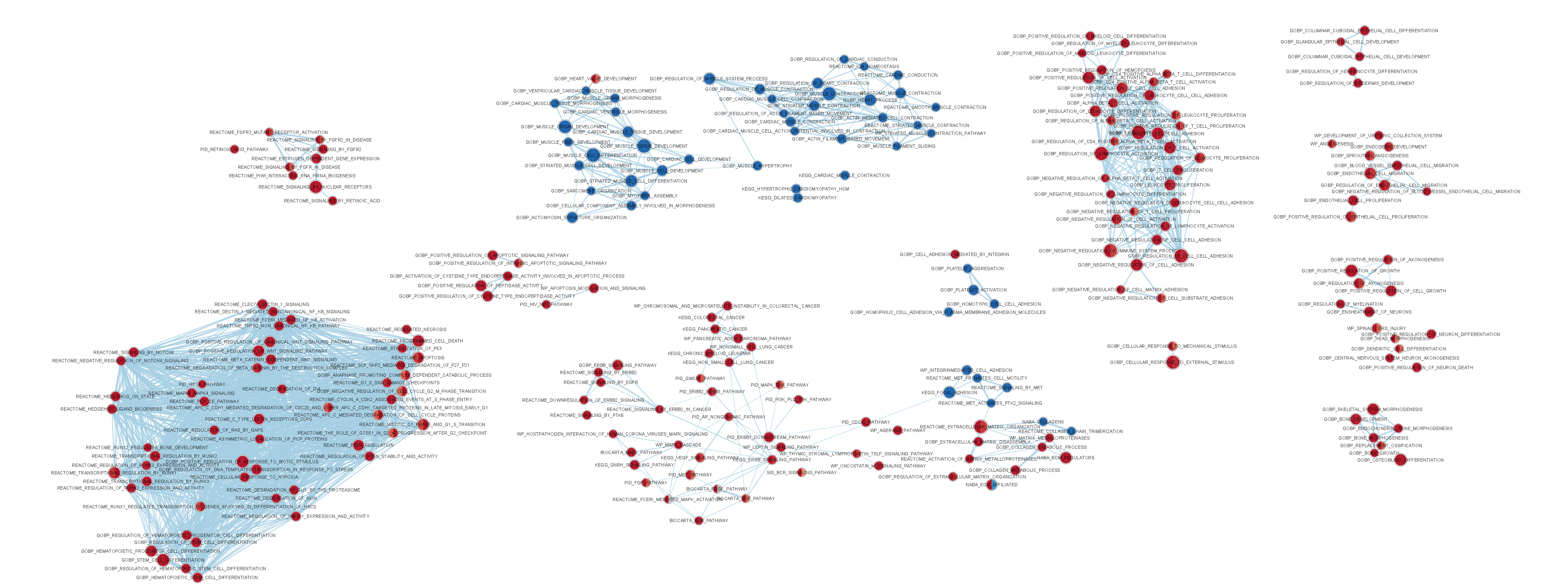

Supplement: Supplementary file 1 [file ijms-23-16037-s001.zip › Data/Figure S2.png]
